# The influence of *H. pylori* infection in HER2-positive gastric cancer cell lines: insights from Wnt/β-catenin pathway

**DOI:** 10.3389/fimmu.2025.1550651

**Published:** 2025-06-26

**Authors:** Valli De Re, Mariateresa Casarotto, Giulia Brisotto, Stefania Zanussi, Mariangela De Zorzi, Ombretta Repetto, Elena Muraro, Paola Spessotto, Paolo Baldo, Vito Racanelli, Marco Vincenzo Lenti, Marino Venerito, Matteo Fassan, Agostino Steffan, Stefano Realdon, Renato Cannizzaro

**Affiliations:** ^1^ Immunopathology and Cancer Biomarkers Unit, Centro di Riferimento Oncologico di Aviano (CRO), IRCCS, Aviano, Italy; ^2^ Molecular Oncology Unit, Centro di Riferimento Oncologico Aviano, (CRO) IRCCS, Aviano, Italy; ^3^ Pharmacy Unit, Centro di Riferimento Oncologico di Aviano (CRO), IRCCS, Aviano, Italy; ^4^ Centre for Medical Sciences, University of Trento and Internal Medicine Division, Santa Chiara Hospital, Provincial Health Care Agency (APSS), Trento, Italy; ^5^ Department of Internal Medicine and Medical Therapeutics, University of Pavia, Pavia, Italy; ^6^ First Department of Internal Medicine, Fondazione IRCCS Policlinico San Matteo, Pavia, Italy; ^7^ Department of Gastroenterology, Hepatology and Infectious Diseases, Otto-von-Guericke University Hospital, Magdeburg, Germany; ^8^ Department of Medicine, Surgical Pathology Unit, University of Padua, Padua, Italy; ^9^ Veneto Institute of Oncology, IOV-IRCCS, Padua, Italy; ^10^ Division of Oncological Gastroenterology, Centro di Riferimento Oncologico di Aviano (CRO) IRCCS, Aviano, Italy; ^11^ Department of Medical, Surgical and Health Sciences, University of Trieste, Trieste, Italy

**Keywords:** *Helicobacter pylori*, trastuzumab, HER2, gastric cancer, Wnt, PD-L1/PD-L2, MSH6, TP53

## Abstract

**Introduction:**

The impact of *H. pylori* infection on the efficacy of trastuzumab in HER2-positive gastric cancer (GC) remains poorly understood, despite growing evidence that tumor microenvironment and host-pathogen interactions influence therapeutic outcomes. This study aimed to investigate how *H. pylori* strains of differing virulence, one high (HV-HP) and one low (LV-HP), affect GC cell behavior, particularly in the context of *ERBB2* (HER2) amplification and Trastuzumab (TRAS)-resistance.

**Methods:**

We used the HER2-amplified NCI-N87 GC cell line, alongside four non-HER2-amplified cell lines (AGS, SNU-1, SNU-16 and SNU-5), to examine the impact of infection. TRAS-resistant derivative cells (N87R) were generated by gradual exposure of the sensitive parental N87 cells (N87p) to increasing TRAS concentrations. Both N87R and N87p cells were infected with HV-HP and LV-HP strains and then treated with epidermal growth factor (EGF), TRAS or a combination of both. The infection was confirmed by confocal microscopy and downstream effects of gene expression were evaluated, focusing on *Wnt-β-catenin* signaling genes linked to metastasis and survival in HER2+ GC. HER2, PD-L1 and PD-L2 protein levels were assessed in all cell lines using multicolor flow cytometry (FACS) before and after HV-HP exposure.

**Results:**

Our data revealed that HV-HP infection reduced *MSH6* mRNA expression, which is indicative of impaired DNA repair, and up-regulated *PDCD1LG2*, suggesting enhanced immunosuppression. FACS analysis showed that HV-HP modulated PD-L2 expression in HER2-amplified N87 cells and to a lesser extent in SNU-16 and SNU-1 cells, while EGF administration increased PD-L1 expression. A strong correlation was observed between ERBB2 expression and TP53, but it was independent of HV-HP. A reduction of CDH1/SNAI ratio was associated with TRAS-resistance in N87 cells.

**Discussion:**

These results suggest that virulent *H. pylori* in cell lines may contribute to altering tumor phenotype by downregulating the DNA repair machinery, and favouring immune evasion by inducing the expression of immunosuppressive signals, such as PDCD1LG2. Moreover, we found that HER2-targeted therapy may contribute to modulation of CD1/immune pathway. Further studies are warranted to determine whether these effects are common in HER2+ GC *in vivo* and whether the coexistence of H. pylori infection and TRAS treatment may influence response to immunotherapy.

## 1 Introduction

*Helicobacter pylori (H. pylori)* infection is a key risk factor for gastric cancer (GC) development (1, 2). The International Agency for Research on Cancer (IARC) classified *H. pylori* as a Group 1 carcinogen in 1994, a classification reaffirmed in 2009. The global incidence of GC varies widely, with the highest age-standardized rates reported in Asia, followed by Latin America, the Caribbean and Europe, and the lowest rates observed in Africa (3).

In 2013 the Japanese government approved national health insurance coverage for antibiotic treatment for *H. pylori* infection in patients with endoscopically diagnosed chronic gastritis. Since then, multiple trials have been conducted to evaluate the efficacy of *H. pylori* eradication in preventing GC in healthy individuals across Colombia, Japan, Korea and several high-risk regions in China.

Epidemiological data suggest that reducing *H. pylori* prevalence could significantly lower GC incidence, particularly in non-cardia subtypes (4, 5). In Italy, guidelines state that *H. pylori* eradication should be carried out in patients with pre-neoplastic gastric lesions, while patients undergoing total gastrectomy should not be treated (6, 7). Moreover, infection’s role may extend beyond tumor initiation, potentially influencing tumor progression and treatment response (8).

Based on overall scientific and clinical evidence, the AIRC Working Group, composed of 35 experts from 20 countries and territories, met in February 2025 as part of the EUROHELICAN project (Accelerating Gastric Cancer Reduction in Europe through *Helicobacter pylori* Eradication).

The geographical variations in GC incidence remain incompletely understood but are likely to be due to risk factors such as *H. Pylori* persistence, genetic susceptibility and lifestyle factors. Furthermore, the long-term co-evolution of *H. Pylori* with human populations has resulted in the emergence of various bacterial strains with different levels of virulence (9). Among *H. pylori* virulent components, the Cytotoxin-associated gene A Pathogenic Island (CagPAI), which includes the oncoprotein cytotoxin-associated gene A (cagA) and the type-IV secretion system (T4SS), plays an important role in host cell modulation, promoting inflammation, epithelial disruption, and oncogenic transformation (10, 11). It is known that CagA delivery occurs at the basolateral, but not on the apical side of the gastric epithelial cells (12), and that this process is facilitated by E-cadherin cleavage. Other virulence factors of *H. pylori*, such as some vacuolating toxin A isofomers (*VacA*) and outer membrane protein (*hom*) haplotypes are also associated with increased pathogenicity and worse clinical outcomes (13). In HER2-positive GC, we have observed that reduced E-cadherin expression correlated with HER2 overexpression and activation of the Wnt/β-catenin pathway, contributing to tumor aggressiveness and poor prognosis (14). E-cadherin normally retains β-catenin at the cell membrane, but its loss enables β-catenin nuclear translocation and transcriptional activation of genes involved in metastasis and survival (15). Notably, both *H. pylori* infection and HER2 signaling have been associated with this pathway, suggesting a possible convergence in tumor progression mechanisms (16–19).

Advanced-stage GC is associated with a poor prognosis, with a 5-year survival rate below 5% in metastatic cases. Standard treatment includes chemotherapy, and in patients with HER2 overexpression, the addition of trastuzumab (20, 21).

HER2, encoded by *ERBB2*, is a member of the *EGFR* receptor family. Unlike other members of the HER family, HER2 does not require ligand binding, but forms heterodimers to initiate downstream signaling. At high levels of expression, HER2 can self-associate to form homodimers (22). Under specific conditions regulated by nucleocytoplasmic transport molecules, homodimers can be translocated to the nucleus (23), where they are involved in yet uncharacterized transcriptional activities (24). However, it is worth noting that HER2 signaling through the Wnt/β-catenin pathway is higher when HER2 is predominantly present in the form of heterodimers (25). HER2 overexpression is a key driver in certain breast and GC, and trastuzumab (TRAS), a monoclonal antibody targeting HER2, is a standard treatment in HER2-positive GC. However, most patients eventually develop resistance to TRAS within one year, and the mechanisms underlying this resistance remain incompletely understood (26).

More recently, immune checkpoint inhibitors (ICIs) have been incorporated into treatment regimens for both HER2-negative and HER2-positive disease, with particularly improved outcomes in patients with PD-L1 positivity tumors (27–30). Despite these advancements, response rate to ICIs vary considerably among patients, likely reflecting differences in anti-tumor immune activity (27). Notably, *H. pylori*-positive status has been associated with better outcomes in this context, pointing to a potential role of *H. pylori* not only in the prevention of GC, but also in treatment response (27). Additionally, molecular investigations have demonstrated that the expression of HER2 and PD-L1 often diverges within tumors, indicating molecular heterogeneity and possible immune escape mechanism (31).

In a previous study, we observed that *H. pylori* plasticity varies between blood donors, first-degree relatives, patients with autoimmune gastritis, and GC patients, suggesting a distinct host-bacterium interaction that may influence tumor behavior and response (32, 33). We also identified a relationship between patient survival, HER2 overexpression, and molecular factors within the WNT/β-catenin pathway, particularly involving E-cadherin, which may be functionally linked to HER2 signaling (14).

However, the potential direct impact of *H. pylori* on HER2-positive tumor cells has not yet been fully investigated.

To address this gap, we explore the effect of *H. Pylori* on NCI-N87 gastric cell line, which harbor ERBB2 gene amplification and constitutive HER2 expression. This study focused on the molecular changes in selected genes previously implicated in the HER2-WNT/β-catenin signaling axis (14), and assesses how these interactions may be influenced by various treatments targeting EGFR/HER2 co-receptor pathways.

## 2 Materials and methods

### 2.1 Cell lines and *H. pylori* strains

To investigate the impact of *H. pylori* on HER2-related signaling and resistance to TRAS in GC, the HER2-amplified, trastuzumab-sensitive NCI-N87 cell line was used (NCI-N87, CRL-5822, ATCC, Manassas, VA, USA), along with four additional HER2-positive gastric cancer cell lines: AGS, SNU-1, SNU-5, and SNU-16 obtained from the American Type Culture Collection. Cells were cultured under standard ATCC protocols. Upon reaching 90% confluence, the cells were detached using TrypLE Express (Life Technologies Corporation, Grand Island, NY, USA), and subsequently expanded in flasks after dilutions as per the manufacturer’s instructions.

The NCI-N87 cell line, derived from a well-differentiated intestinal gastric carcinoma, was isolated in 1976 from a liver metastasis in a male patient prior to cytotoxic therapy (34). The cell line was passed three time through athymic nude mice as a xenograft before being established. It exhibits stable microsatellite status (MSS) and is positive for EGFR, HER2, while moderately positive for HER3 and HER4. The *ERBB2* gene is amplified, and *ERBB2, ERBB3 and ERBB4* genes have several variants as reported in a previous study (35). NCI-N87 cells harbor a homozygous pathogenic mutation in the *TP53* gene (p.Arg248Gln) and a deletion in the transcription factor *SMAD4*.

Four further HER-2 positive ATCC cell lines were used in the experiment: the primary gastric adenocarcinoma AGS cell line (AGS, CRL-1739, ATCC, Manassas, VA, USA), the primary undifferentiated gastric adenocarcinoma SNU-1 cell line (SNU-1, CRL-5971, ATCC, Manassas, VA, USA), the metastatic poorly differentiated gastric adenocarcinoma SNU-5 cell line (SNU-5, CRL-5973, ATCC, Manassas, VA, USA), and SNU-16 cell line (SNU-16, CRL-5974, ATCC, Manassas, VA, USA), isolated from the ascites of a metastatic gastric carcinoma patient. As well as NCI-N87, SNU-5 and SNU-16 cell lines present mutations in TP53 gene, whereas AGS and SNU-5 show mutations in CDH1 gene.

The AGS, SNU-1 and SNU-16 cell lines were cultured in ATCC- formulated RPMI-1640 medium (ATCC 30-2001), with 10% FBS; for the SNU-5 cell line the ATCC-formulated Iscove’s Modified Dulbecco’s Medium (ATCC 30-2005) supplemented with 20% FBS was used. Concerning the adherent AGS cells, upon reaching 90% confluence, they were detached using TrypLE Express (Life Technologies Corporation, Grand Island, NY, USA), and subsequently expanded in flasks after dilutions as per the manufacturer’s instructions. For cells characterized by growth of aggregates in suspension (SNU-1, SNU-5 and SNU-16), the manufacturer’s instructions were followed for the appropriate dilutions. To establish TRAS-resistant N87 cells (N87R), the sensitive parental NCI-N87 cell line (from here on named N87p) was exposed to increasing concentrations of the anti-HER2 receptor TRAS (Ontruzant, Organon Italia, Roma, Italy) as previously reported (36). Briefly, cells were cultivated for 15 days at progressively increasing TRAS concentrations, starting from 15 μg/mL up to 500 μg/mL. Cells were then cultured at the same concentration of 500 μg/mL TRAS for further 60 days. For all subsequent experiments, resistant cells were grown by adding 500 μg/mL TRAS in the culture medium (37). The viability of both the cell types was verified by Propidium Iodide staining (AccuChip Kit, NanoEnTek, Korea) and fluorescence measurement on ADAM-MC automated cell counter (NanoEnTek Inc., Korea).

**Figure 1:**
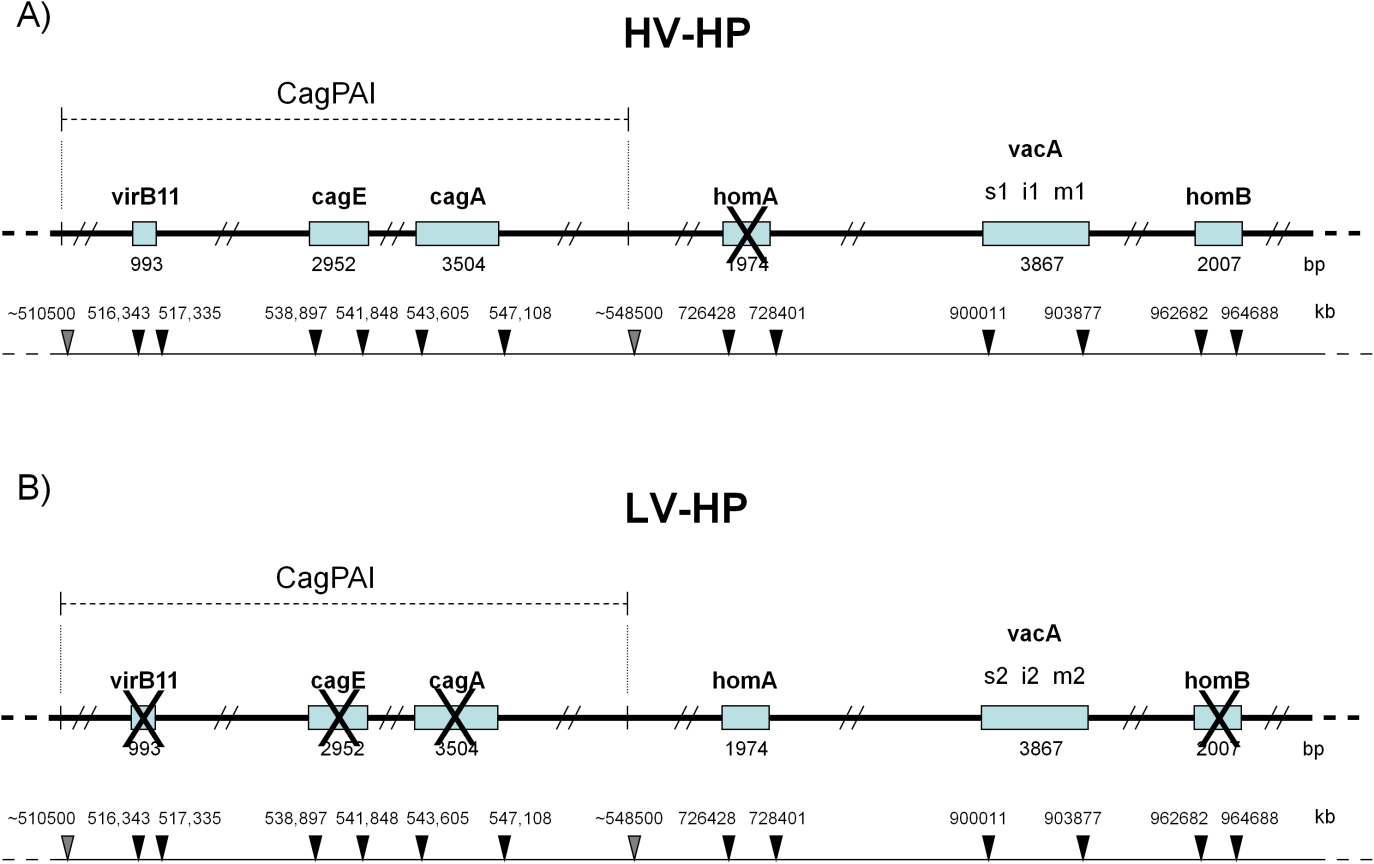
Molecular profiles of *H. pylori* according to virulence factors. **(A)** HV-HP profile (*cagA+cagE+virB11+vacAs1i1m1+homB+)*, defined by the simultaneous presence of *virB11* (located in the left half of the CagPAI), *cagA* and *cagE* (both located in the right half of the CagPAI), the *vacA s1i1m1* haplotype, the presence of the *homB* gene and the absence of the *homA* gene in their loci. **(B)** LV-HP profile (*cagA-cagE-virB11-vacAs2i2m2+homA+)*, defined by the concomitant deletion of *virB11*, *cagA* and *cagE* genes, the *vacA s2i2m2* haplotype, the presence of *homA* gene and the absence of *homB* gene in their loci. The genes of interest are indicated by open rectangles. Crosses represent missing genes. The position of CagPAI and individual genes on the underlying partial physical map of *H.pylori* genome are indicated by grey and black arrows, respectively. The high-virulence strain was characterized by the concomitant presence of a stable Cag pathogenicity island (CagPAI), a vacA s1i1mx genotype, and homB, features associated with a strongly virulent phenotype, whereas the low-virulence strain lacked these markers (33). HV-HP, highly virulent *H. pylori*; LV-HP, low virulent *H. pylori*.

To evaluate the impact of *H. pylori* on the signalling pathway under study, two different *H. pylori* strains were chosen based on some previously tested virulence factor (33): *VirB11*, *CagE* and *CagA*, which are located at the beginning, middle and end of the CagPAI island; the absence of these genes was considered as a poorly functioning CagPAI (33, 34). *H. pylori* strains were also examined for *VacA* isoforms and *HomA/B* loci. The *VacA s1i1m1* haplotype encodes a VacA protein isoform with a higher vacuolization capacity than *VacA s2m2i2*. The *homB* haplotype confers a higher level of pathogenicity than *homA*. The first strain, named highly virulent *H. pylori* (HV-HP), exhibited the putative highly virulent molecular profile *cagA* ^+^ *cagE^+^virB11^+^vacAs1i1m1^+^homB^+^*, while the second one, called lowly virulent *H. pylori* (LV-HP), displayed the presumed less virulent molecular profile *cagA* ^-^ *cagE^-^virB11* ^-^ *vacAs2i2m2^+^homA^+^* (**Figure 1**).

Both the strains were recovered from a -80°C ultra-low temperature refrigerator and revitalized by culturing them on Pylori Selective Medium plates (Bio-Mérieux, Florence, Italy) according to standard *H. pylori* culture conditions (38). After 3 to 5 days of incubation at 37°C in a microaerophilic environment generated by sachets (Oxoid, Basingstoke, UK), the bacteria were plated on Columbia Sheep Blood Agar (Kima, Padua, Italy), and further incubated for 3 days under the same conditions. This process was repeated twice to expand the bacterial growth. On the day of co-culture, the grown bacteria were confirmed as *H. pylori* through Gram-negative staining, curved or spiral shape, and positivity for oxidase, catalase and urease testing. Hence, bacteria were collected from the agar plates and resuspended in liquid medium (RPMI-1640 without FBS). The concentration of the resulting suspension was quantified using a spectrometer (Thermo Electron Corporation, Cambridge, UK). For AGS, SNU-1, SNU-5 and SNU-16 cell lines, only HV-HP strain was used to evaluate the bacterial impact on the signaling pathway under study; the co-culture was carried out for all cell lines as above described above. The five HV-HP co-cultured cell lines and their controls without bacteria were collected separately and then analyzed by flow cytometry to evaluate PD-L1 and PD-L2 expression on the cells surface.

### 2.2 Design of the study

**Figure 2:**
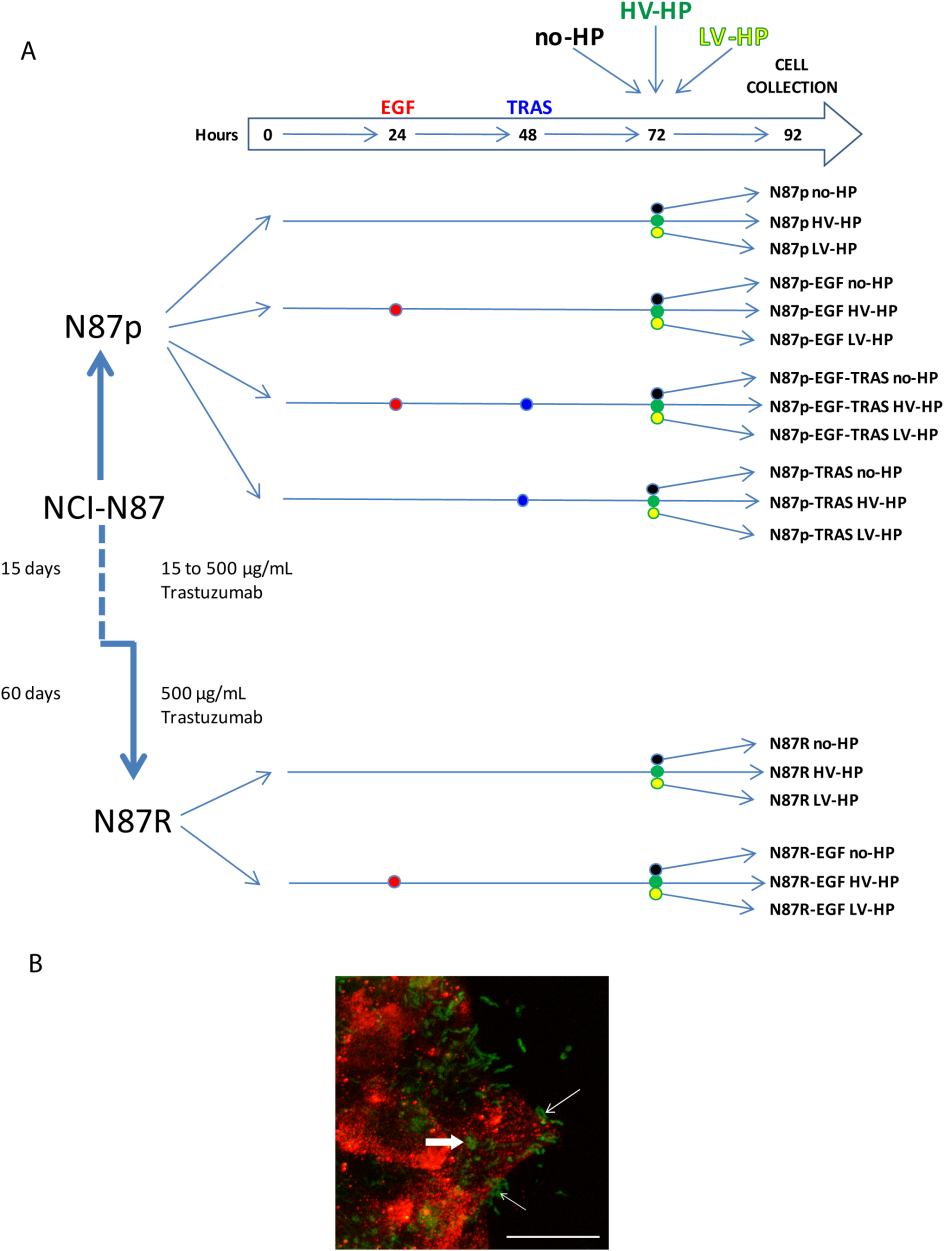
**(A)** Summary of the study design, including planned treatment. p, parental; R, resistant; EGF, Epidermal Growth Factor; TRAS, Trastuzumab; no-HP, no infection with *H. pylori*; HV-HP, highly virulent *H. pylori;* LV-HP, lowly virulent *H. pylori*;. **(B)** Representative image of confocal microscopy immunofluorescence of NCI-N87 cells infected with HV-HP. N-cadherin staining (red) was used to visualize the interaction of cells with Five-(and 6-) carboxyfluorescein diacetate, succinimidyl ester (CFDA-SE) stained bacteria (green). Thin white arrows indicate that bacteria interact with the cells near the plasma membrane; bold white arrow indicates bacteria inside the cells. Scale bar = 20 μm.

The experiment design is resumed in **Figure 2A**. EGF is instrumental for studying HER2, as it activates the EGFR pathway, which can form both homodimers and heterodimers with HER2. The formation of heterodimers is particularly important, especially in the context of HER2 overexpression. Given that HER2 lacks a known direct ligand, EGF serves as a valuable tool for exploring the activated Wnt pathway and its interaction with HER2. Additionally, EGF helps create conditions that mimic the tumour microenvironment, enhancing our understanding of HER2’s role in tumor progression within our experiment.

In detail, N87p and N87R cells were seeded into 12-wells flat-bottom plates to comply with the following conditions: i) culture medium without any addition (N87p; N87R); ii) culture medium with 100 ng/mL EGF (Bio-techne, Minneapolis, MN, USA) from hour 24 (N87p-EGF; N87R-EGF); iii) culture medium with 100 ng/mL EGF from hour 24 followed by the addition of 250 µg/mL TRAS from hour 48 (N87p-EGF-TRAS); iv) culture medium with 250 µg/mL TRAS from hour 48 (N87p-TRAS). After 72 hours, co-cultures were established by adding HV-HP or LV-HP with at a multiplicity of infection of 100:1 to the wells containing cells treated as described above. Co-culture, and its controls without bacteria, was carried out for 20 hours. Cells from each treatment condition were then collected separately and stored at -80°C for subsequent mRNA extraction, and gene and protein expression analyses.

### 2.3 Confirmation of NCI-N87 and AGS cell lines *H. pylori* infection by immunofluorescence

N87p and AGS cell lines were grown on glass coverslips to 80-90% confluence and co-cultured with 5(and 6)-carboxyfluorescein diacetate, succinimidyl ester (CFDA-SE) (Molecular Probes) labelled bacteria as described below. Bacteria collected from agar plates were pelleted (3000xg for 5’), washed, resuspended in 3ml sterile 1x PBS and incubated with 5μM CFDA-SE at 37°C for 20 minutes. After two washes with sterile 1x PBS, the bacterial culture was standardized spectrophotometrically at 600 nm and added to the glass coverslips containing the expanded cells. After 20 h, the coverslips were fixed with 4% paraformaldehyde (PFA) for 10 min, blocked with 2% FCS and 1% BSA (Sigma) for 1 h at room temperature (RT) and incubated with specific primary antibodies at 4°C overnight.

Anti β1 integrin (clone EP1041Y, Abcam, 1:200 dilution) or anti E-cadherin (36/E cadherin, BD, 1:50 dilution) antibodies for AGS cells and anti N-cadherin (ab 18203, Abcam, 1:100 dilution) antibodies for NCI-N87 cells were used. Cells were incubated with the appropriate Alexa Fluor^®^ 568-conjugated secondary antibodies (Life Technologies) for 1 hour at RT and counterstained with TO-PRO™ (Life Technologies) to visualize the nuclei. All solutions and washes were conducted in 1x PBS. Samples were then mounted with a glycerol-based antifade agent. Images were acquired using a confocal scanner system (TCS SP8 FSU AOBS, Leica Microsystems), equipped with a Leica DMi8 inverted microscope (Leica Microsystems). The maximum projections obtained were then processed using LAS software (Leica Microsystems) and Volocity 3D image analysis software (PerkinElmer).

### 2.4 RNA extraction and quantification

RNA extraction was carried out using the RNeasy Plus Micro kit (Qiagen, Hiden, Germany) following the manufacturer’s protocol. Subsequently, RNA quantification was performed using both Nanodrop (Thermo Fisher Scientific, Wilmington, DE, USA) and the Qubit™ RNA HS Assay (Life Technologies Corporation, Eugene; OR, USA) on the Qubit fluorometer 2.0. instrument (Invitrogen, MA, USA). Furthermore, RNA quality assessment was performed using the High Sensitivity RNA Screen Tape kit (Agilent, Santa Clara, CA, USA) on the 2200 Tape Station system (Agilent, Santa Clara, CA, USA).

### 2.5 Nanostring nCounter gene expression analyses

NanoString gene expression analyses were performed using a customized panel based on the results of our previous study (14), which included all the genes listed in **Supplementary Table 1**. For each sample, a total of 300ng of RNA was hybridized with reporter and capture probes following the manufacturer’s protocol provided by NanoString Technologies (Seattle, WA). Then, the RNA-probes complexes were bound on a nCounter Cartridge using the nCounter Prep Station. RNA counts were then performed by scanning 555 fields of view with the nCounter Digital Analyzer (NanoString Technologies, Seattle, WA, USA). Raw data analyses were carried out using the Advanced nSolver Analysis Software. We considered genes with a log2 fold change (Log_2_FC) threshold of ≥ |0.38| and a p-value of <0.05 to be differentially expressed. For further analysis, we identified the most highly upregulated genes as those with a Log2 fold change value greater than |0.5|. This Log2FC cut-off point was chosen based on the distribution of expression values within the dataset, in order to exclude genes demonstrating minimal, biologically irrelevant variation.

### 2.6 Flow cytometry

The following antibodies were employed in flow cytometry analyses: Brilliant Violet 421 (BV421) mouse anti-human HER-2 antibody (IgG1, 24D2, BioLegend), Phycoerythrin (PE) mouse anti-human PD-L1 antibody (IgG1, MIH1, Becton Dickinson [BD]), Allophycocyanin (APC) mouse anti-human PD-L2 antibody (IgG1, MIH18, BD). Properly labeled isotypic antibodies were used as negative controls (BV421 mouse IgG1 [X40, BD]; PE mouse IgG1 [MOPC-21, BD]; APC mouse IgG1 [MOPC-21, BD]). All antibodies were used in an appropriate volume of BD^®^ CellWASH Fluid (Becton, Dickinson and Company, NJ, USA), incubated 30 minutes at room temperature in the dark, washed, and re-suspended in an appropriate volume of BD^®^ CellWASH Fluid. At least 10^4 cells were collected during sample acquisition (excluding cell doublets and debris). Flow cytometry analysis was performed with the BD FACSCanto™ II Flow Cytometer (BD Biosciences, CA, USA) belonging to the flow cytometry core facility of our Institute. Photomultiplier voltages and compensation were set up with unstained and stained cells. Flow cytometry data were analyzed with BD FACSDiva™ Software (Becton, Dickinson and Company BD Biosciences, CA, USA), and FlowJo (Tree Star, Ashland, OR, USA) software.

### 2.7 Protein extraction and immunoblotting

Proteins were extracted from frozen N87, SNU-1 and SNU16 cells. Briefly, 2x10^6 cells of each frozen sample was homogenized and lysed (4°C, 15 min) in 60 μL Lysis Buffer (Thermo Fisher Scientific) containing Universal Nuclease (Thermo Fisher Scientific), 1x protease and phosphatase inhibitor cocktail (Thermo Fisher Scientific) and 0.1% (w/v) RapiGest SF (Waters). The lysates were subjected to two cycles of freezing (-80°C) and thawing (4°C) and sonication (2x 60s). The tubes were centrifuged at 8,000 x g for 20 min to remove membrane debris. Protein concentration was measured using the Pierce BCA Protein Assay Kit (Thermo Fisher Scientific). Protein extracts were stored at − 80°C until analysis.

Protein (20μg per sample group) was fractionated on 4-15% Criterion TGX Stain-Free gels (Bio-Rad). Gel images were acquired with the Chemidoc system (Bio-Rad, Hercules, CA, USA) to document equal protein loading among samples. Proteins were then electrotransferred to nitrocellulose membranes (Cytiva, Uppsala, Sweden) and probed with the following primary antibodies: CDH1 (1:1000; #124198, GeneTex) HER2/ErbB2 (29D8) (1:1000; #2165, Cell Signalling Technology), p53 (DO-1) (1:1000; #sc-126, Santa Cruz Biotechnology), PD-L1 (1:500; #PA520343, Thermo Scientific), PD-L2 (D7U8C) (1:500; #82723, Cell Signalling Technology), Snail (C15D3) (1:1000; #3879, Cell Signalling Technology). Primary antibodies were detected with HRP-conjugated goat anti-rabbit and anti-mouse IgG Fc fragment antibodies and Super-Signal West Femto maximum sensitivity substrate (Thermo Scientific). Blots were imaged using the Chemidoc system (Bio-Rad).

### 2.8 Statistical analysis

Comparison of mRNA expression levels between different treatment groups or *H. pylori* strains co-culture was evaluated considering at least three independent experiments and using using t-tests and ANOVA for two groups, and Kruskal-Wallis, and Mann-Whitney tests for multiple groups. P values were adjusted for multiple comparisons using the method of Bonferroni to control for the false discovery rate. Correlation between treatment conditions and dependent variables such as TRAS resistance or *H. pylori* infection were assessed using the Spearman rank correlation analysis. Volcano plots were used to show highly specific mRNAs of interest. Differential gene expression analysis from volcano plot was performed using Advanced nSolver Softwares and VolcaNoseR (39). High-dimensional Wnt/β-catenin-associated mRNA expression data were stratified into distinct treatment groups using Principal Component Analysis (PCA), visualized through scatterplots, and validated with ANOVA tests. All analyses were performed by R software version 4.2.2 and MedCalc version 22.021. For flow cytometry analyses, the mean fluorescence intensity (MFI) of isotypic controls was subtracted from the MFI of specific antibodies (HER2, PD-L1 and PD-L2) analysed in 3 different experiments. Significant differences between H. pylori treated and untreated samples were assessed by Student’s T-test for each cell line tested (i.e. 3 different experiments). *P-*value of < 0.05 was considered statistically significant (*P < 0.05, **P < 0.01, ***P < 0.001).

## 3 Results

### 3.1 The influence of *H. pylori* infection on GC cell lines

**Figure 3:**
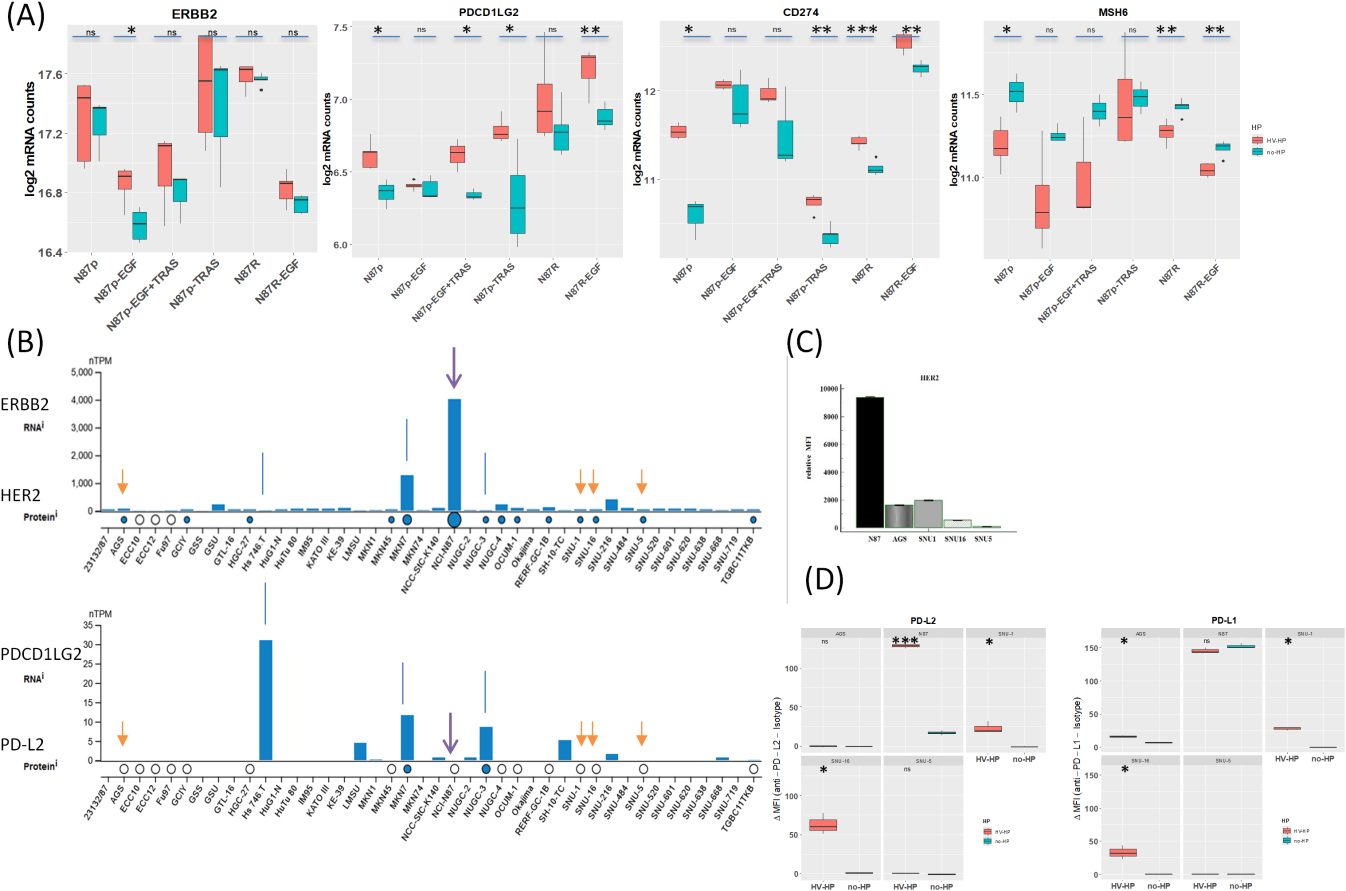
**(A)** Summary of gene expression changes in N87 gastric cancer cells infected with H. pylori. While *ERBB2* levels remain stable *upon Helicobacter pylori* infection in N87p gastric cancer cells., *PDCD1LG2 and CD274* is upregulated and *MSH6* downregulated, highlighting infection may drive modulation of immune and genomic stability pathways. The increase of *PDCD1LG2* is significant in most treated cells, except in N87p-EGF and trastuzumab-resistant N87R cells, where the increase did not reach a statistical significance. H. pylori infection reduces *MSH6* mRNA levels in N87p gastric cancer cells, as well as in TRAS-resistant cells (N87R and N87R-EGF), suggesting impairment of DNA repair or stress-response pathways. Statistical significance (Student’s t-test): P < 0.05 (*), P < 0.01 (**), P < 0.001 (***). **(B)**. Public dataset: Human Protein Atlas (HPA). Basal expression of HER2 and PD-L2 in gastric adenocarcinoma cell lines from the Human Protein Atlas (HPA) (40). RNA expression is shown as normalized transcripts per million (nTPM) for each cell line. Protein expression based on mass spectrometry data is indicated by circles. Data confirm constitutive *ERBB2*/HER2 expression in NCI-N87 cells and variable levels in other cell lines. White circles indicate absence of PD-L2 protein detection in N87, AGS, SNU1, SNU16, and SNU5 cells. Arrows mark the cell lines analyzed in the present study. **(C)**. HER2 expression assessed by flow cytometry.N87p cells displayed a markedly higher HER2 fluorescence signal, as indicated by the median relative fluorescence intensity (MFI), compared to the other analyzed cell lines (AGS, SNU1, SNU16, SNU5). Median relative MFI = 2700 ± SEM1605. **(D)** PD-L2 expression assessed by flow cytometry. Boxplots ahow relative MFI values are shown for uninfected and HV-HP infected cell lines (AGS, N87, SNU-1, SNU-16 and SNU-5). Asterisks indicate statistically significant increases in median MFI in HV-HP-infected cells compared to uninfected controls in N87 (P < 0.001), SNU-16, and SNU-1 (p<0.05), as determined by Student’s *t-*test (performed in R).

To determine whether *H. pylori* infection affects the *Wnt* pathway in HER2+ gastric tumors, we first co-cultured N87p cells with two different *H. pylori* strains previously isolated from the gastric biopsies of two patients with GC (33). The HV-HP strain was considered highly virulent due to the presence in its genome of the CagPAI containing *virB11, cagE*, and *cagA* genes, and of *vacAs1i1m1* haplotype and *homB* gene, while the LV-HP was classified as low virulent, since it showed CagPAI deletions, *vacA s2i2m2* haplotype and *homA* profile associated with GC, as previously described (33). We selected the N87 cell line, derived from a metastatic, well-differentiated intestinal GC, as a valuable model for assessing the therapeutic effectiveness of the TRAS drug in cases with *ERBB2* gene amplification. We obtained ERBB2 gene expression mRNA and protein data from the Human Protein Atlas (HPA) (https://www.proteinatlas.org) for commonly used gastric cell lines. The data were used in accordance with the CC BY-SA 3.0 licence, with reference to the original publication (40). According to the HPA, only the NCI-N87 and MKN7 cell lines harbour ERBB2 amplification and constitutively express HER2 (**Figure 3B**). Molecular details of the NCI-N87 cell line are provided in the Material and Methods section. To explore the changes associated with the HER2 context, we also investigated the effects of HV-HP in AGS, SNU-1, SNU-5, and SNU-16 cell lines. Using FACS and Western blot analyses, we confirmed high HER2 expression in the N87p cell line and low levels in the remaining cell lines (**Figure 3C**; **Supplementary Figures S1**, **S2**). The expression levels were categorised as either high or low, depending on whether the relative median fluorescence intensity (MFI) of all cell lines was greater than or less than 2.700. Confocal microscopy verified the *H. pylori* infection in our experimental study (N87 in **Figure 2B**, AGS in **Supplementary Figure S3**).

Initial analysis revealed significant differences in the expression of several Wnt/β-catenin-related genes (Log2FC ≥ |0.38|, p-value < 0.05) when comparing the mRNA levels of *H. pylori-*infected and uninfected N87p cells (**Supplementary Figure S4**). In contrast, infection with the low-virulence LV-HP strain did not induce any statistically significant changes in gene expression at the same Log2FC threshold (**Supplementary Figure S4B**). As only the more virulent HV-HP strain, but not the LV-HP strain, affected the expression of genes in the Wnt/β-catenin pathway during co-culture, all subsequent analyses were conducted using data from HV-HP strain co-cultures.

Notably, the *MSH6* (mutS homolog 6), *CD274* and *PDCD1LG2* genes showed the most significant and striking fold changes following HV-HP infection. *MSH6*, essential for DNA repair, was under-expressed, while *CD274* and *PDCD1LG2*, both associated with suppression of T-cell activation, were over-expressed in HV-HP-infected cells.

At FACS analysis, all five cell lines were negative for PD-L2; however, after HV-HP infection, three cell lines (N87, SNU-1 and SNU-16) showed significantly increased PD-L2 expression compared to their uninfected counterparts (P<0.05; **Figure 3D**; **Supplementary Figure S1**), with N87 showing the highest ΔMFI level. PD-L2 was not observed in any cell line by immunoblotting (data not shown).To further determine whether HV-HP modulates *PDCD1LG2* gene expression in a HER2-dependent or independent manner, we assessed the Pearson correlation between *ERBB2* and *PDCD1LG2* mRNA levels under different conditions. As summarized in **Table 1**, HV-HP–infected N87 cells, but not non-infected controls, showed a statistically significant positive correlation between ERBB2 and *PDCD1LG2* expression (t = 3.284, p = 0.0463). This correlation was not observed in other cell lines, which highlights the impact that genetic composition has on this phenotype. Although *ERBB2* and *PDCD1LG2* correlation is modest (p = 0.0463), it supports the hypothesis that *ERBB2* signaling may contribute to PD-L2 regulation in the context of HV-HP infection in HER2+ N87 cell line. However, data is not robust (p = 0.0463) and warrants further investigation to be conclusive.

**Table 1:** Pearson correlation between ERBB2 levels and the gene mRNA shown on the table under different conditions.

Gene	HP	Treatment	Coefficient	T-value	P-value	R-squared	Significance
PDCD1LG2	HV-HP	none	0.308	3.284	0.0463	0.782	*
PDCD1LG2	none	N87R	-0.858	-3.187	0.0498	0.772	*
CD274	none	none	-1.618	-25.965	0.0245	0.999	*
CDH1	HV-HP	none	-0.329	-3.812	0.0318	0.829	*
MSH6	HV-HP	none	-0.43	-3.498	0.0395	0.803	*
MSH6	HV-HP	EGF	-1.201	-4.581	0.0445	0.913	*
SNAI1	none	N87R	1.874	5.57	0.0114	0.912	*
TP53	HV-HP	N87R	1.393	6.619	0.00702	0.936	**
TP53	HV-HP	none	1.282	11.364	0.00146	0.977	***
TP53	HV-HP	EGF	1.371	8.021	0.0152	0.97	*
TP53	HV-HP	TRAS	0.935	9.574	0.0107	0.979	*
TP53	none	N87R	1.089	4.291	0.0233	0.86	*
TP53	none	none	1.413	13.192	0.0482	0.994	*
TP53	none	EGF	1.522	4.99	0.00755	0.862	**
TP53	none	TRAS	0.822	15.859	0.000545	0.988	***

Significance * p<0.05, ** p<0.01, *** p<0.005.

Following HV-HP infection, an increase in PD-L1 protein expression could not be confirmed by FACS or Western blot analysis in N87p (**Figure 3**; **Supplementary Figures S1**, **S2**). The uninfected cell line already exhibited PD-L1 expression. The other cell lines, SNU-1 and SNU-16 showed a modest increased PD-L1 expression after HV-HP infection (ΔMFI<50, **Figure 3D**).

With regard *MSH6*, the difference between uninfected and HV-HP-infected cells was only evident in untreated and trastuzumab-resistant (N87R) cells (**Figure 3A**). *MSH6* expression varies among GC cell lines and showed a modest negative correlation with *ERBB2* expression under HV-HP infection in the N87 cell line (t-value= –3.498, p = 0.0395; **Table 1**). According to TCGA data from the HPA, *MSH6* expression above a defined threshold (cut-off: 9.86 transcripts per million) is associated with reduced five-year survival in GC patients (32% versus 48% in the low-expression group), though this difference is not statistically significant (p = 0.33). In our patient cohort from a previous study [n = 55; (41)], in which the majority of cases (75%) exhibited a microsatellite stable (MSS) phenotype, 21 patients (32.3%) exhibited *MSH6* mRNA levels above the median. No significant difference in *MSH6* expression frequency was observed between HP-positive and HP-negative cases. However, a larger sample size is required to draw definitive conclusions.

### 3.2 Wnt signaling is upregulated following EGF stimulation and partially reduced after trastuzumab administration

To investigate Wnt signal transduction at the level of EGFR and HER2 receptors, we evaluated the differential expression of selected Wnt-related mRNA genes following engagement with specific mediators, i.e. EGF, TRAS and both EGF and TRAS, compared to the untreated cells.

EGF is particularly useful for studying HER2 because it activates the EGFR pathway, which helps EGFR to form dimers with HER2. Heterodimer formation is particularly critical in cases of HER2 overexpression. Additionally, EGF creates conditions that mimic the tumor microenvironment, enhancing our understanding of HER2’s role in tumor progression.

**Figure 4:**
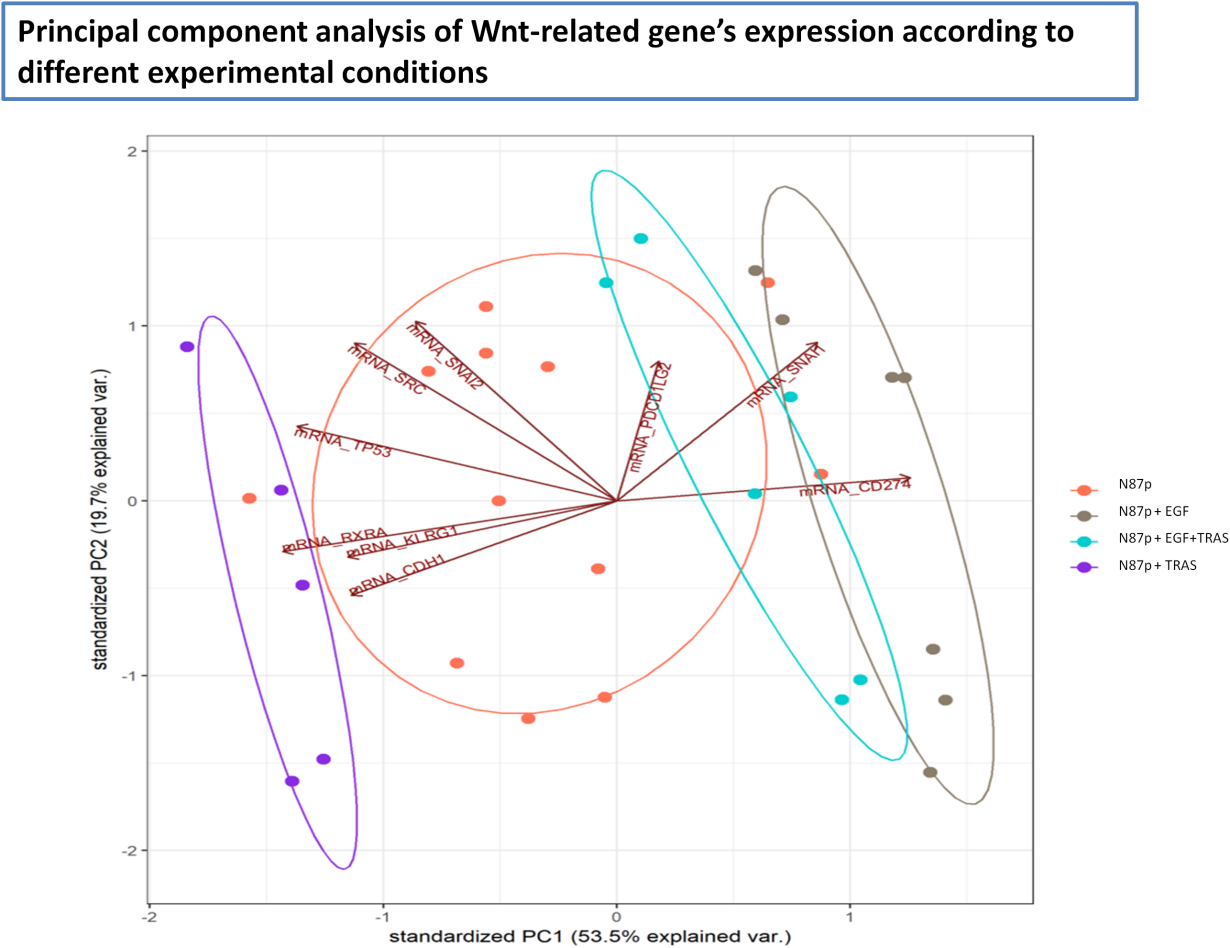
Principal Component Analysis (PCA) of Wnt pathway-related mRNA expression in N87p cells. PCA score plot of mRNAs differentially expressed in untreated and treated N87p cells. Genes were selected based on differential expression analysis related to the Wnt/β-catenin pathway (volcano plot analysis). Red dots represent untreated N87p cells; grey and purple dots correspond to cells treated with EGF and TRAS, respectively; light blue dots indicate cells treated with both EGF and TRAS. Vectors indicate the contribution of each mRNA to the variance explained by the first two principal components (PC1 and PC2). Confidence ellipses, automatically generated by NanoString nSolver software, illustrate group clustering based on expression profiles.

Principal Component Analysis (PCA) of Wnt/β-catenin pathway-related gene expression revealed four distinct clusters corresponding to untreated, EGF-treated, TRAS-treated, and EGF+TRAS-treated N87p cells (**Figure 4**). The separation of these groups along both PC1 and PC2 suggests treatment-specific transcriptional profiles. Notably, TRAS-treated samples were positioned toward the negative end of PC1, whereas EGF-treated cells clustered along the positive axis of PC1. The addition of TRAS to EGF treatment resulted in a reduction of the PC1 score, shifting the samples toward more intermediate values. Untreated cells were clearly separated from all treated conditions.

### 3.3 Changes in the differentially expressed gene pattern following EGF treatment

**Figure 5:**
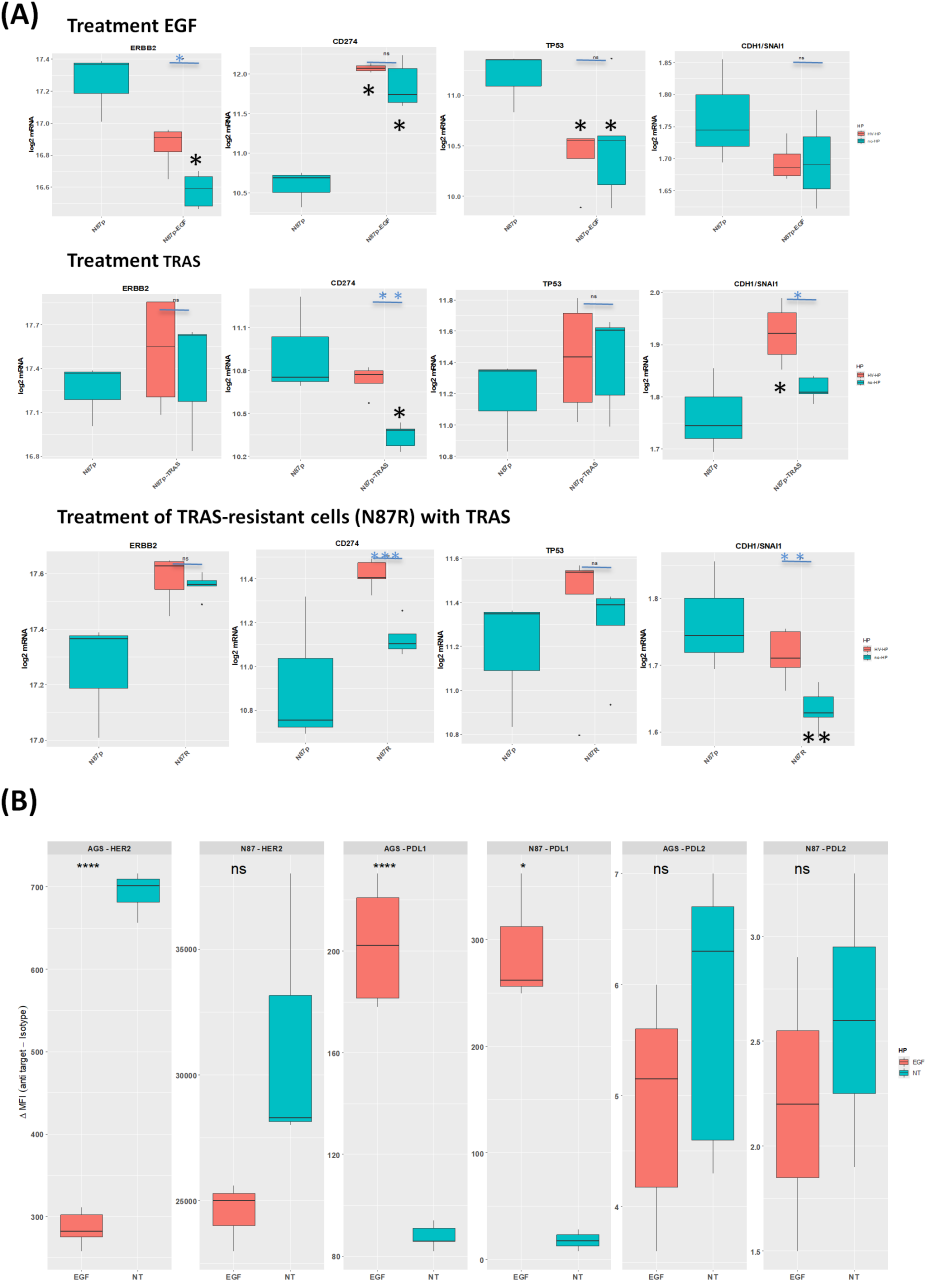
Effects of EGF and TRAS treatments on gene expression and surface marker levels in gastric cancer cell lines. **(A)** Boxplots showing mRNA expression levels (log_2_ normalized counts) of selected genes *ERBB2*, *TP53*, and *CDH1/SNAI1*, measured by NanoString technology in AGS, N87, and TRAS-resistant N87R cells following treatment with EGF or TRAS. Two types of statistical comparisons are indicated: light blue asterisks denote significant differences between HV-HP-infected and uninfected treated cells, while larger black asterisks indicate significant differences between treated conditions and the untreated, uninfected N87p control. **(B)** Boxplots showing the median fluorescence intensity (MFI), calculated as the signal from anti-target antibody minus isotype control, for HER2, PD-L1, and PD-L2. Data were obtained by flow cytometry (FACS) in AGS and N87 cells under untreated (NT) and EGF-treated conditions. Data represent the mean of three independent replicates (*n* = 3). Asterisks indicate statistically significant differences as determined by Student’s *t*-test in R (*p* < 0.05; p < 0.01; *p* < 0.001; ns, not significant).

In EGF-treated N87p cells, the most significant changes included the over-expression of *CD274*, along with reduced *ERBB2* and *TP53* mRNA levels (Log2FC ≥ |0.5|, p<0.05; **Supplementary Figure S5**; **Figure 5**). Based on mRNA expression analysis, PD-L1 showed significantly altered protein levels. Although less pronounced, HER2 expression also changed following EGF treatment in both N87 and in AGS cells lines (**Figures 5A, B**).

In N87 cells, TP53 expression significantly decreased following EGF treatment (**Figure 5A**). TP53 mRNA levels showed the strongest correlation with ERBB2, but this correlation was independent of HV-HP infection (**Table 1**, **Figure 5A**). This association was further validated using transcriptomic data from one our previous GC patient cohort (r = 0.22, p = 0.001; (41).

HV-HP infection attenuated the effect of EGF treatment on ERBB2 expression in N87 cells only (**Figure 5A**, p<0.05).

### 3.4 Changes in the differentially expressed gene pattern following TRAS treatment

Among the differentially expressed genes in TRAS-sensitive N87p cells initially defined using a threshold of Log2FC ≥ |0.38| and p-value < 0.05, we observed that the highest Log2FC value in TRAS treatment alone resulted in a reduction in *CD274* expression (**Supplementary Figure S6a** *Log2FC* ≥ |0.48|). However, subsequent infection of N87p-TRAS with HV-HP partially reversed this effect, leading to increased expression of *CD274* (**Figure 5A**, p<0.01).

Interestingly, HV-HP infection was also associated with a reduction in *CTNNB1* (catenin beta 1), and *SNAI1* mRNA expression levels (**Supplementary Figure S6B**). Given that SNAI1 acts as a transcriptional repressor of CDH1, and that CTNNB1 contributes to mesenchimal signaling, the downregulation, along with stable or increased expression of CDH1, results in an elevated CDH1/SNAI1 expression ratio (**Figure 5A**, p<0.05). This shift indicates a suppression of the EMT and a reinforcement of epithelial characteristics. Moreover, we observed an inverse correlation between ERBB2 and CDH1 expression, which disappeared following TRAS treatment (**Table 1**). This suggests that TRAS, may influence E-cadherin expression in the N87 cells line by modulating HER2. We did not investigate this effect in cell lines expressing low levels of HER2.

Collectively, these findings suggest that, while TRAS alone exerts a beneficial effect by reducing CD274 expression, HV-HP infection counteracts this immune-related effect by upregulating CD274 and *PDCD1LG2*. At the same time, HV-HP appears to enhance the anti-EMT activity of TRAS by down regulated CTNNB1 and SNAI, thereby promoting a more epithelial-like transcriptional profile, as reflected by the increased CDH1/SNAI1 ratio.

### 3.5 Wnt-related genes associated with TRAS resistant N87R cells

To explore *H. pylori*’s potential role in the molecular mechanism underlying *TRAS* resistance, we first compared gene expression profiles between TRAS-resistant N87R cells and their TRAS-sensitive parental N87p counterparts through pairwise analysis of volcano plot (**Supplementary Figure S7Aa**). Differentially expressed genes were initially defined using a threshold of Log2FC ≥ |0.38| and p-value < 0.05. Among these, the four most upregulated genes (i.e.*PDCD1LG2*, *ZEB1(*zinc finger E-box binding homeobox 1), *SNAI2*(Snail f*amily tr*anscriptional repressor 2, also known a*s slu*g), and *SNAI1*(Snail family transcriptional repressor 1) displaying Log2FC > 0.5 were considered the most prominently altered in TRAS-resistant N87R cells. These genes are known to be involved in Epithelial-to-Mesenchymal Transition (EMT), in part through the modulation of E‐cadherin (*CDH1)* expression.

Furthermore, although both N87p and N87R cells were treated with TRAS, decreased expression of *CD274 and PDCD1LG2* was only observed in N87p-TRAS cells (**Figure 5A**). Conversely, decreased expression of *CDH1/SNAI1* was observed in N87R cells (**Figure 5A**). These results suggest that genes associated with CDH1/SNAI expression ratio may play a significant role in determining TRAS resistance than the TRAS treatment itself.

In TRAS-resistant N87R cells,HV-HP infection associated with a decreased in MSH6 and an increased in CD274 expression (**Figure 3A**). We did not assess TRAS-resistance in HER2-low cell lines, as this was beyond the scope of the present study.

## 4 Discussion

Trastuzumab (TRAS), direct against HER2, is approved as a first-line treatment in combination with chemotherapy for patients with HER2-positive metastatic or unresectable GC. Increasing evidence also highlights the role of *H. pylori* infection in both the pathogenesis and treatment outcomes of GC (4, 5, 8, 42–45). In a cohort of patients treated at our institution, *H. pylori* was found in 93 out of 311 GC samples (29.9%), regardless of tumor stages (TNM I-II: 20.7%, TNM III-IV: 30.3%). Previous studies, in which *H. pylori* prevalence exceeded 50%, have shown that *H. pylori* eradication significantly reduced the risk of GC development and metachronous GC, suggesting a long-term protective effect (42–45). Furthermore, a recent study indicated that treating *H. pylori* may improve survival in GC patients with the infection (8). Despite these findings, the impact of *H. pylori* on the efficacy of TRAS therapy stays unclear.

Our results demonstrated that infection with a highly virulent (HV-HP) strain significantly increases PD-L2 mRNA and protein expression in HER2-overexpressing N87 cells (**Figure 3**). This effect was not observed in uninfected cells or those infected with a low-virulent LV-HP strain (**Figure 3D**). PD-L2 is generally not expressed in GC cell lines (**Figure 3D**). However, we found its evidence of its expression in both SNU-1 and SNU-16 following HV-HP infection, albeit at a lower ΔMF1 than in N87 cells (**Figure 3D**). Notably, up-regulation occurred even in the absence of immune cells, suggesting a direct response by tumor cells themselves. In N87p cells treated with TRAS-, HV-HP infection further induced PDCD1LG2 expression (**Figure 5A**). TRAS-resistant cells were also associated with an increase in PDCD1LG2 expression (**Figure 5A**). By contrast, EGF stimulation was found to selectively up-regulated the CD274 gene and its PD-L1 protein, but not PD-L2, in both the N87 and AGS cell lines (**Figures 5A, B**). PD-L1 was detectable in N87p at baseline, and neither CD274 mRNA nor protein expression changed following *H. pylori* infection, except in N87p cells treated with TRAS (**Figure 3A**; **Supplementary Figure 1**). In these cells, HV-HP increased expression. Conversely, treatment with TRAS of non infected cells, led to a reduction in CD274 and PD-L1 expression (**Figure 5**).

These findings indicated that, while restricted to certain cells lines, HV-HP infection and the modulation of EGFR/HER2 signaling can may modulate PD-L1 and PD-L2 expression, thereby contributing to tumor immune evasion. However, further studies are needed to confirm this effect in humans. While PD-L1 has been extensively investigated, PD-L2 remains largely unexplored. Immunohistochemical analyses have confirmed PD-L2 expression in GC tissues, with higher levels observed in patients with distant metastases (46, 47). Previous studies have shown that *H. pylori* infection, particularly with CagA-positive strain, can increase PD-L1 expression in both *in vitro* models and in human gastric tissues (48–54). More recently, up-regulation of PD-L2 has also been reported, as *H. pylori* appears to activate key inflammatory pathways that are associated with PD-L2 expression (55, 56).

Similarly, HER2 inhibition can reshape the tumor microenvironment and modulate the expression of immune checkpoint ligands such as PD-L1 and, to a lesser extent, PD-L2. *In vitro* studies have demonstrated that TRAS-sensitive GC recruit PD-L1-positive immune cells and up-regulated PD-L1 in HER2-amplified GC cell lines co-cultured with peripheral blood mononuclear cells (57–59). Moreover, PI3K/AKT and MAPK pathways, which are frequently activated by HER2 overexpression (60, 61), promote the expression of both PD-L1 and PD-L2 (62, 63). One *in vitro* study found higher PD-L1 expression in TRAS-resistant HER2-positive GC cells compared to their parental counterparts, and blocking PD-L1 reversed the TRAS resistance in these cells (64).

These findings imply that HER2‐positive GC, particularly those that are immunologically “hot”, but under suppression *via* the PD‐1/PD‐L1 axis, may benefit from TRAS treatment, as was demonstrated in the KEYNOTE‐811 trial (27). However, data on PD-L2 expressions in GC patients remain limited, as most clinical investigations have focused on PD-L1. Further studies are warranted to elucidate the effect of PD-L2, especially in patients with metastatic HER2-positive and PD-L1-negative GC.

Moreover, EGF-driven EGFR activation also up-regulated CD274, suggesting a role for HER family signaling in promoting immunosuppression within the tumor microenvironment. (**Figure 5**). Immune checkpoint blockade (ICB) therapy using anti PD-1 monoclonal antibodies has shown modest efficacy (11.9% response rate) in advanced GC (28, 65). Combined HER2-targeted and ICB therapies have demonstrated improved outcomes and may overcome TRAS resistance (66–69). Although PD-L2 is less studied than PD-L1, it is co-expressed in a significant proportion of GC cases and is associated with clinical stage, tumor progression and poor prognosis (56, 70, 71). Notably, some PD-L1 negative GC patients still respond to anti-PD1 treatment, possibly due to PD-L2 involvement (65). Emerging evidence also implicates long-non coding RNAs (lncRNA) and tumor–derived exosomes in regulating PD-L2, emphasizing its potential as a prognostic biomarker in GC (71, 72). However, the mechanisms regulating PD-L2 expression remain poorly understood. Our study contributes to filling this gap by revealing differential modulation of PD-L1 and PD-L2 in response to *H. pylori* infection and TRAS treatment, highlighting the importance of EGFR/HER2 signaling interaction. Although EGF has not been identified as a prognostic factor in GC, elevated levels have been associated with poor overall survival in several studies (73–77). The co-expression of EGFR and HER2 has been linked to more aggressive tumor behavior (78). Collectively, our data provide insights into the molecular mechanisms supporting the therapeutic benefit of targeting HER2/EGFR heterodimers rather than HER2/HER2 homodimers, a recent focus of drug development efforts. These findings also highlight the potential impact of *H. pylori* infection in the context of combined HER2/ICB therapy (79).

We also explored the impact of HV-HP on Wnt/β-catenin signaling and on DNA repair pathways, both of which were associated with worse prognosis in HER2-positive metastatic GC patients (14). We focused on 30 genes from the Wnt/ß-catenin signaling we previously found to be differentially expressed, evaluating their expression following administration of EGF, TRAS, or their combination, and comparing these with untreated cells and *H. pylori*-infected cells. EGF, which activates the EGFR pathway and can form both homo- and heterodimers with HER2, serve as a useful tool for exploring HER2. Since HER2 lacks a known ligand, EGF helps to simulate EGFR stimulation *in vivo* within the tumor microenvironment and aiding in the study of Wnt pathway’s interaction with HER2.

HV-HP infection led to down-regulation of *MSH6*, a crucial gene involved in mismatch repair, in both TRAS-sensitive and -resistant cells (**Figure 3A**; **Supplementary Figure 2B**). This finding suggests that, in addition to its immunomodulatory effects, *H. pylori* may disrupt the DNA repair machinery, thereby promoting genomic instability. Previous studies have established a link between reduced DNA repair capacity and an increased risk of GC in individuals infected with *H. pylori*, a situation exacerbated by oxidative stress and inflammation caused by the bacterial infection (80). Notably, by the age of 85, the cumulative risk of malignant transformation was reported to be 45.5% in individuals with both mutations in DNA repair genes and *H. pylori* infection, compared to 14.4% for those without the infection (58). Our data supports this model, indicating that HV-HP may suppress *MSH6* expressions, potentially increasing microsatellite instability (MSI), which is a recognized predictive biomarker for GC prognosis and response to ICB therapy and chemotherapy (81–85). Additionally, the *H. pylori* CagA protein may inhibit the translocation of the tumor suppressor protein BRCA1, a gene involved in recombination repair, from the cytoplasm to the nucleus (86). Consequently, *H. pylori* may impair DNA repair trough both *MSH6* and *BRCAI*, contributing to chemotherapy resistance even in the absence of gene mutations, a phenotype reminiscent of “BRACness” (86). Importantly, MSI is associated with an enhanced cytotoxic response to DNA-damage agents like paclitaxel, and this effect is further amplified when combined with TRAS (87, 88). Of interest, inhibitors of poly (ADP-ribose) polymerase (PARP), an enzyme that play a key role in the DNA damage response by adding branched PAR chains to recruit other repair proteins, have emerged as a promising therapeutic approach for GC patients (89–91).

Moreover, N87 cells harbor *TP53* mutations and microsatellite instability (MSI) features. In our study, HV-HP did not further modulate *TP53*. TP53 was well correlated with HER2 expression, and EGF-treated N87 cells showed reduced *TP53* mRNA levels (**Table 1**, **Figure 5A**). Although p53 degradation by H. pylori CagA has previously been reported (92, 93), our findings indicate additional indirect modulation of *TP53* via EGR/HER2 signaling independent of *H. pylori* infection. Notably, we observed an opposite modulation of *TP53* levels in TRAS-treated sensitive N87p cells compared to EGF-treated cells (**Figure 5A**).

We also observed a decrease in the CDH1/*SNAI1 ratio* in TRAS-resistant N87R cells that were not infected, and the effect was counteracted by TRAS treatment of sensitive N87p cells (**Figure 5A**)*. SNAI1* is an EMT regulator that promotes tumour invasiveness by repressing *CDH1*/E-cadherin transcription. *C*onsistent with literature, increased of *SNAI* and reduction of *CDH1* are linked and both associated with poor prognosis in HER2-positive GC (14, 94, 95).Based on our data this effect appeared to be more closely associated with TRAS-resistance (**Figure 5A**).

In summary, our study showed that both HV-HP infection and the modulation of EGFR/HER2 may contribute to the up-regulation of PD-L2. In addition HV-HP was found to suppresses *MSH6*, which could potentially increase genomic instability. These effects were particularly evident in the HER2-amplified N87 cell line in the presence of virulent HV-HP strains, which suggests that also the genetic background of the cell and of the bacteria contributes. In addition EGF-driven EGFR activation enhances PD-L1. Together, our findings suggest that *H. pylori* and activation of EGF/HER2 signaling increase the aggressiveness of GC and are consistent with recent clinical data indicating that *H. pylori* eradication could provide long-term survival advantage for GC patients. However, it is noteworthy that the presence of *H. pylori* and EGF/HER2 activation, which up-regulates PD-L1/PD-L2 expression and downregulating DNA repair machinery, has contributed to the identification of more specific GC subsets that are potentially responsive to combination of chemo-immunotherapies. Further studies are warranted to establish whether these effects are prevalent in HER2+ GC *in vivo*, and whether the co-existing H. pylori infection and TRAS treatment influences response to immunotherapy.

## Data Availability

The original contributions presented in the study are publicly available. This data can be found here: 10.5281/zenodo.14598616.

## Supplementary material

Supplementary Figure 1 Surface expression analysis of HER2, PD-L2, and PD-L1 in GC cell lines. **(A)** Surface levels of HER2, PD-L2, and PD-L1 in GC cell lines (N87, AGS, SNU-1, SNU-16, and SNU-5) after 20 hours of co-culture with a highly virulent *Helicobacter pylori* strain (HV-HP), compared to untreated controls (NT). Mean fluorescence intensity (MFI) from isotype controls was subtracted from the signal obtained with specific antibodies (PD-L1 and PD-L2). Significant differences between HV-HP and NT conditions were assessed for each cell line based on the mean of three independent replicates using Student’s *t*-test. **(B)** Surface expression of HER2, PD-L1, and PD-L2 in the N87 and in AGS cell lines after 48 hours of EGF treatment, compared to untreated controls.

Supplementary Figure 2 Immunochemical detection of five selected proteins, indicated by gene names on the right. Protein samples represent pools extracted from three GC cell lines across nine different experimental groups. **(a)** Image of the SDS-PAGE gel acquired using the Chemidoc system before to transfer onto nitrocellulose membranes. **(b)** Western blots analysis of the selected proteins.

Supplementary Figure 3 Representative confocal immunofluorescence images of AGS cells infected with HV-HP (green). AGS cells were immunostained for β1 integrin (**A**, red) or E-cadherin (**B**, red). In panel A, the maximum projection (right) and the xyz projections (left) highlight the predominant intracellular localization of *H. pylori*. Panel B shows E-cadherin expression levels before **(a)** and after **(b)** HV-HP infection, with bacteria observed in close association with the plasma membrane. Scale bar = 20 μm.

Supplementary Figure 4 Differential expression of Wnt/β-catenin-related genes in N87p cells upon *Helicobacter pylori* infection. **(A)** Volcano plot showing the differential expression of selected Wnt/β-catenin-related genes in N87p cells infected with a highly virulent *H. pylori* strain (HV-HP) compared to uninfected N87p cells. (**B)** Volcano plot showing the differential expression of Wnt/β-catenin-related genes in N87p cells infected with a low-virulence *H. pylori* strain (LV-HP) compared to uninfected controls. Genes with an absolute log_2_ fold change (|log_2_FC|) ≥ 0.38 and a *p*-value ≤ 0.05 were considered significantly modulated. Significant genes are labeled next to their corresponding data points. HV-HP = highly virulent *H. pylori* strain; LV-HP = low-virulence *H. pylori* strain.

Supplementary Figure 5 Differential mRNA expression in EGF-treated N87p cells with or without *Helicobacter pylori* infection. **(A)** Volcano plot showing differentially expressed genes between EGF-treated and untreated N87p cells. **(B)** Volcano plot comparing EGF-treated N87p cells with versus without infection by a highly virulent *H. pylori* strain (HV-HP). **(C)** Volcano plot comparing EGF-treated, HV-HP-infected N87p cells to untreated and uninfected controls. Red and blue dots represent significantly upregulated and downregulated genes, respectively (*p* ≤ 0.05 and |log_2_FC| ≥ 0.2).

Supplementary Figure 6 TRAS treatment and HV-HP infection of TRAS-sensitive N87p cells. **(A)** Volcano plot of differentially expressed genes in TRAS-treated versus untreated N87p cells. **(B)** Volcano plot of HV-HP infected, TRAS-treated N87p compared to their uninfected counterpart. Red and blue dots indicate significantly upregulated and downregulated genes, respectively (p-value ≤ 0.05 and |log_2_FC| ≥ 0.2).

Supplementary Figure 7 Wnt-related genes associated with TRAS-resistance N87R cells, with or without HV-HP infection. **(A)** Volcano plot analysis of differentially expressed Wntβ-catenin-related genes in TRAS-resistant N87R versus TRAS-sensitive N87p cells. **(B)** Volcano plots highlighting differentially expressed genes in HV-HP infected N87R cells compared to uninfected N87p cells. **(C)** Volcano plots of differentially expressed genes in TRAS-resistant N87R with versus without HV-HP infection. **(D)** Volcano plots of differentially expressed genes in N87R and N87p cells, both treated with TRAS.Volcano plots show genes with an absolute log_2_ fold change (|log_2_FC|) ≥ 0.2 and p-value ≤ 0.05. The names of significantly modulated genes are labeled. HV-HP = highly virulent *H. pylori* strain.

Supplementary Figure 8 Effects of EGF administration in TRAS-resistant N87R cells compared to TRAS-sensitive N87p cells. **(A)** Volcano plot comparing EGF-treated N87R cells to untreated, TRAS-sensitive N87p cells (without HV-HP infection). **(B)** Volcano plot comparing EGF-treated N87R cells to untreated N87p cells in the presence of HV-HP infection. Red and blue dots indicate significantly upregulated and downregulated genes, respectively (p-value ≤ 0.05 and |log_2_FC| ≥ |≥ 0.2).
