# Supplementary figures and images for: The influence of H. pylori infection in HER2-positive gastric cancer cell lines: insights from Wnt/β-catenin pathway
Source: Front Immunol. 2025 Jun 26;16:1550651. doi: 10.3389/fimmu.2025.1550651 (PMC12240786; doi:10.3389/fimmu.2025.1550651)

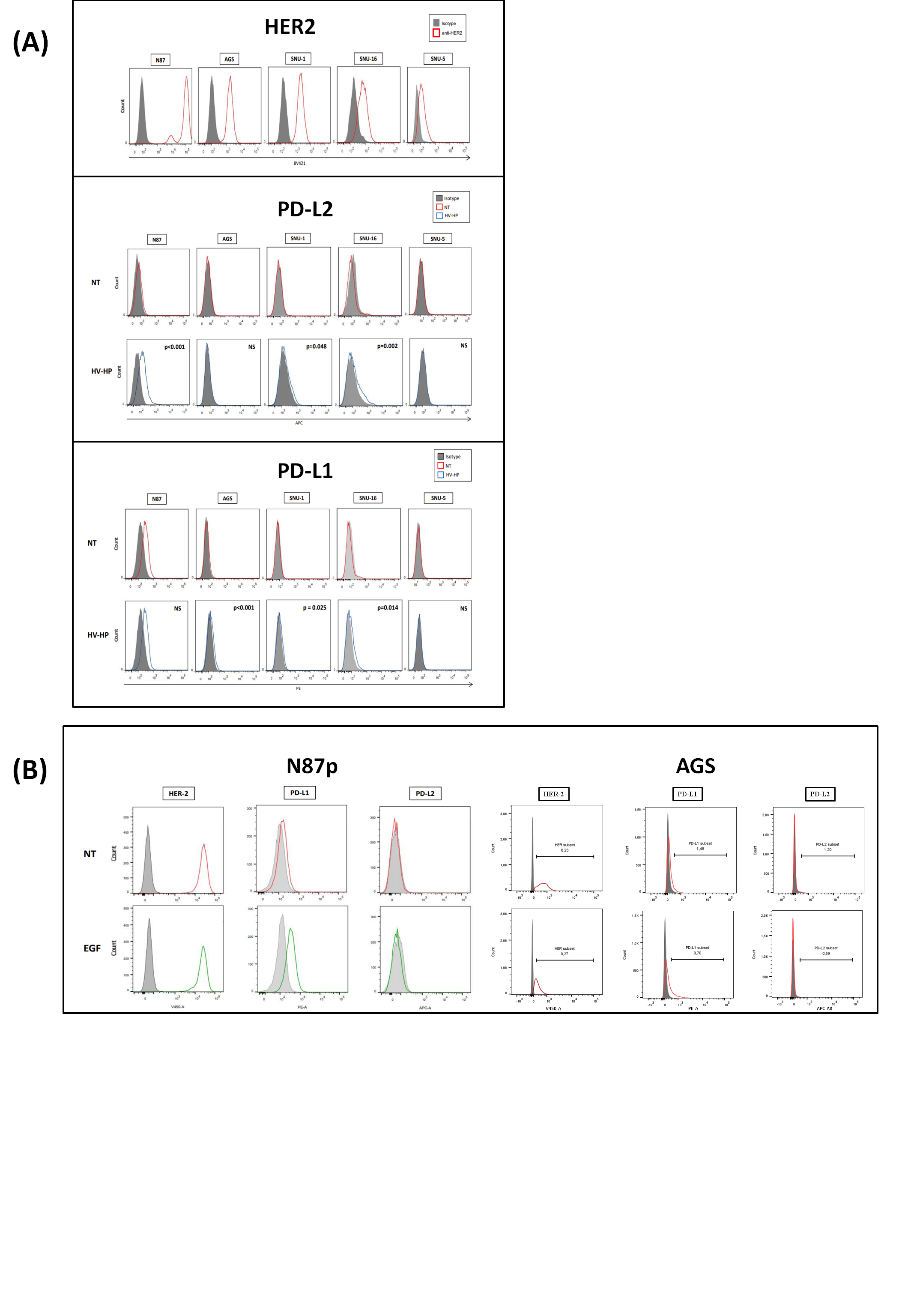

Supplement: Supplementary Figure 1 — Surface expression analysis of HER2, PD-L2, and PD-L1 in GC cell lines. (A) Surface levels of HER2, PD-L2, and PD-L1 in GC cell lines (N87, AGS, SNU-1, SNU-16, and SNU-5) after 20 hours of co-culture with a highly virulent Helicobacter pylori strain (HV-HP), compared to untreated controls (NT). Mean fluorescence intensity (MFI) from isotype controls was subtracted from the signal obtained with specific antibodies (PD-L1 and PD-L2). Significant differences between HV-HP and NT conditions were assessed for each cell line based on the mean of three independent replicates using Student’s t-test. (B) Surface expression of HER2, PD-L1, and PD-L2 in the N87 and in AGS cell lines after 48 hours of EGF treatment, compared to untreated controls. [file Image1.tif]

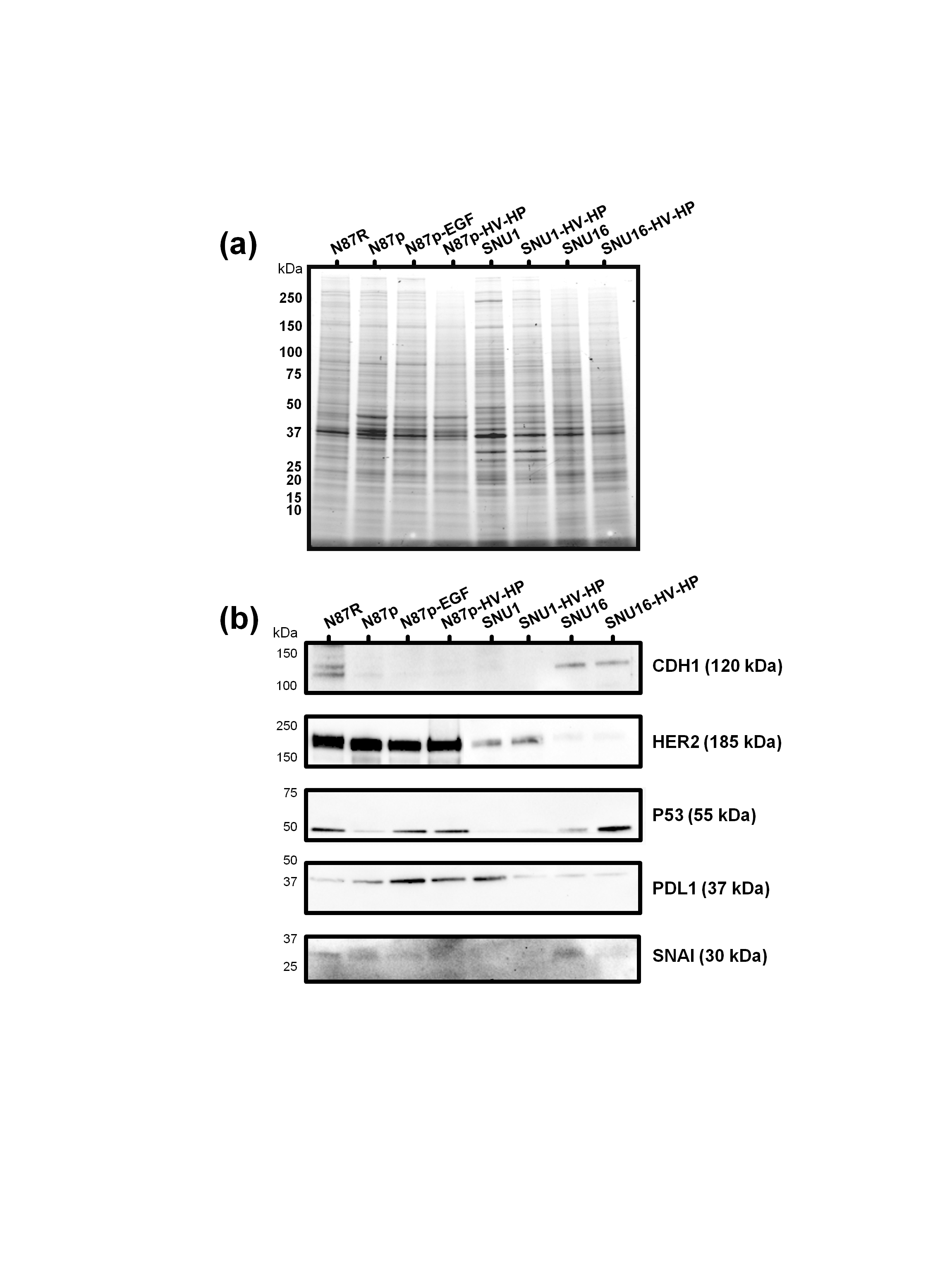

Supplement: Supplementary Figure 2 — Immunochemical detection of five selected proteins, indicated by gene names on the right. Protein samples represent pools extracted from three GC cell lines across nine different experimental groups. (a) Image of the SDS-PAGE gel acquired using the Chemidoc system before to transfer onto nitrocellulose membranes. (b) Western blots analysis of the selected proteins. [file Image7.tif]

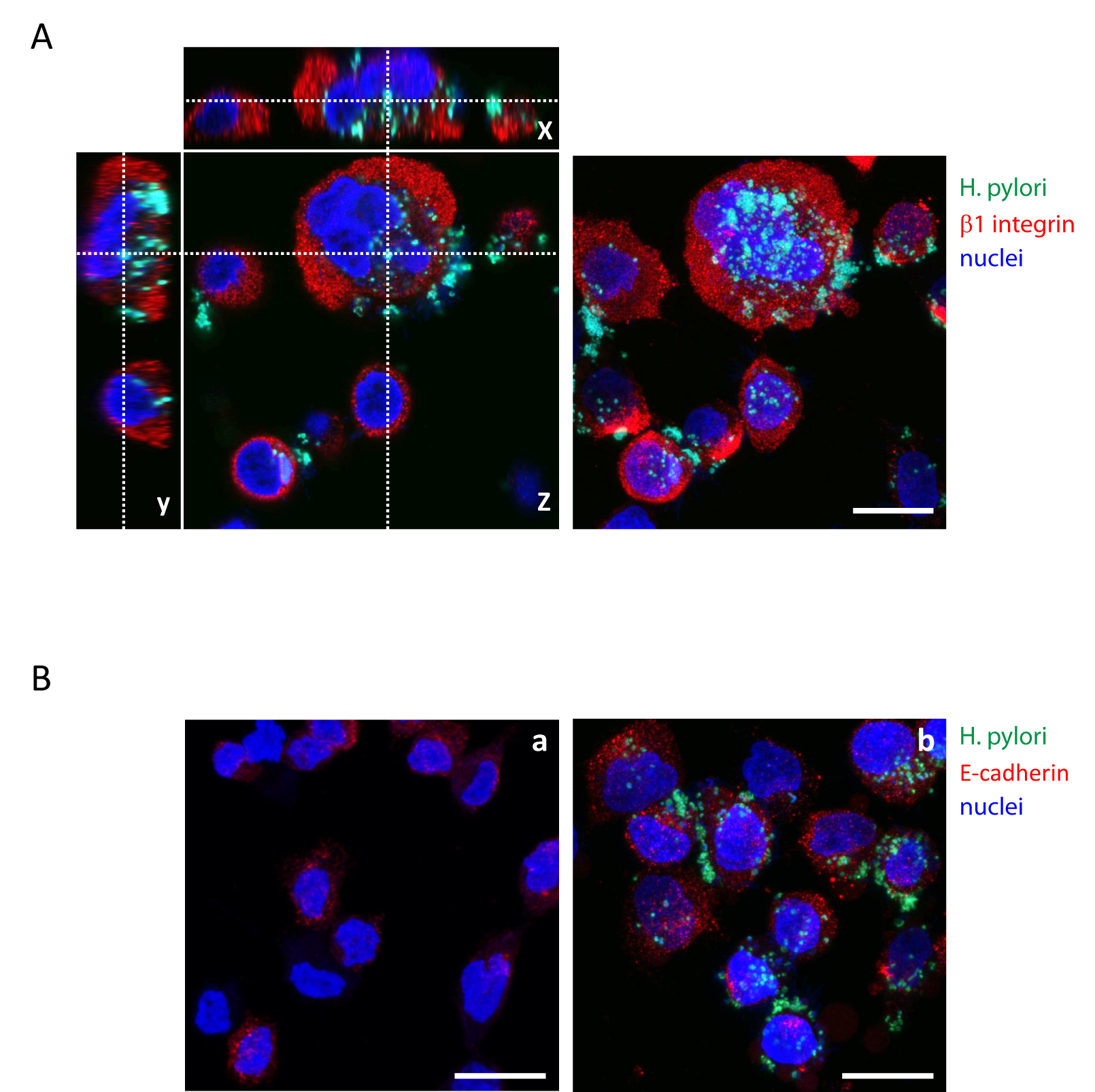

Supplement: Supplementary Figure 3 — Representative confocal immunofluorescence images of AGS cells infected with HV-HP (green). AGS cells were immunostained for β1 integrin (A, red) or E-cadherin (B, red). In panel A, the maximum projection (right) and the xyz projections (left) highlight the predominant intracellular localization of H. pylori. Panel B shows E-cadherin expression levels before (a) and after (b) HV-HP infection, with bacteria observed in close association with the plasma membrane. Scale bar = 20 μm. [file Image8.tif]

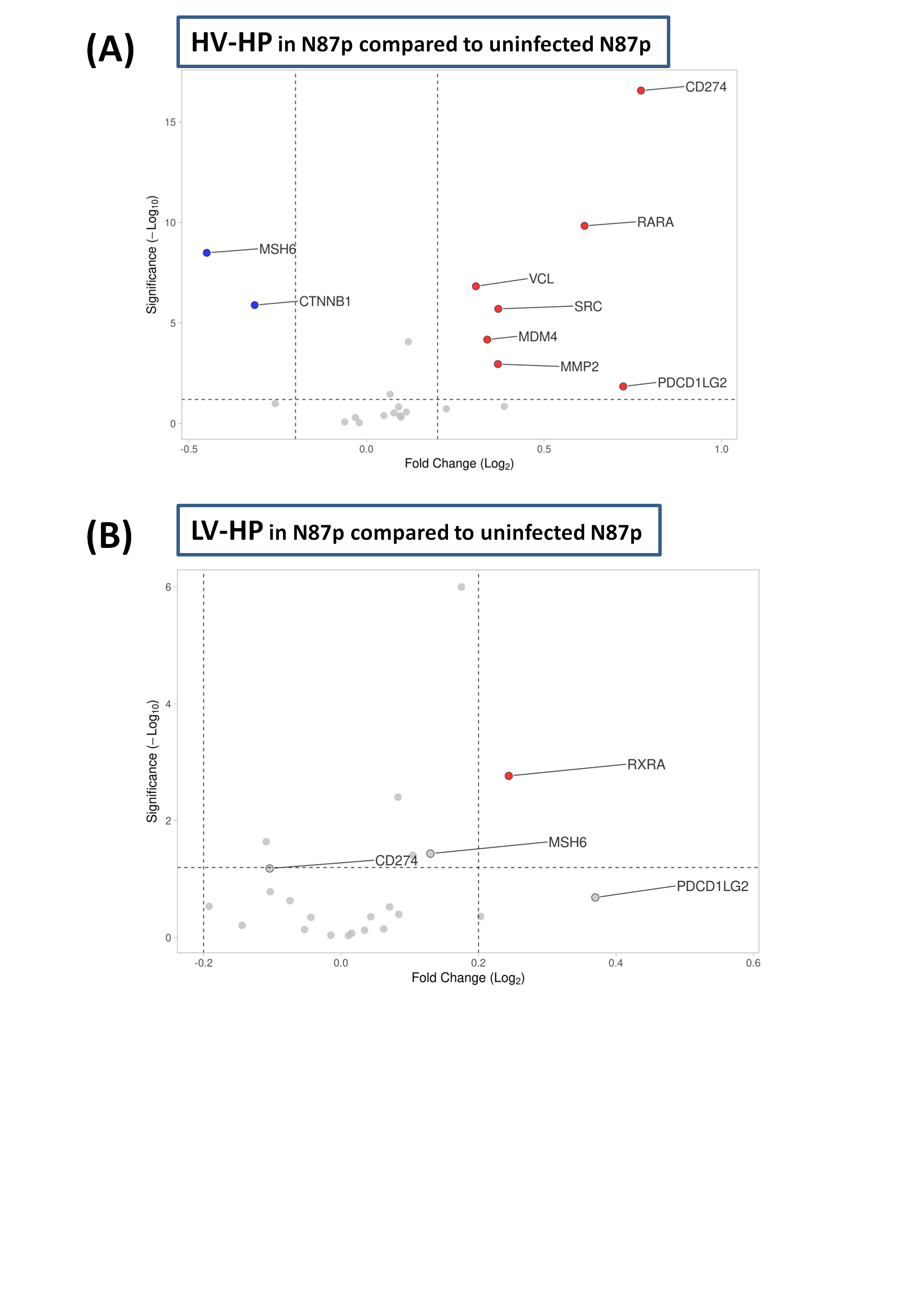

Supplement: Supplementary Figure 4 — Differential expression of Wnt/β-catenin-related genes in N87p cells upon Helicobacter pylori infection. (A) Volcano plot showing the differential expression of selected Wnt/β-catenin-related genes in N87p cells infected with a highly virulent H. pylori strain (HV-HP) compared to uninfected N87p cells. (B) Volcano plot showing the differential expression of Wnt/β-catenin-related genes in N87p cells infected with a low-virulence H. pylori strain (LV-HP) compared to uninfected controls. Genes with an absolute log2 fold change (|log2FC|) ≥ 0.38 and a p-value ≤ 0.05 were considered significantly modulated. Significant genes are labeled next to their corresponding data points. HV-HP = highly virulent H. pylori strain; LV-HP = low-virulence H. pylori strain. [file Image9.tif]

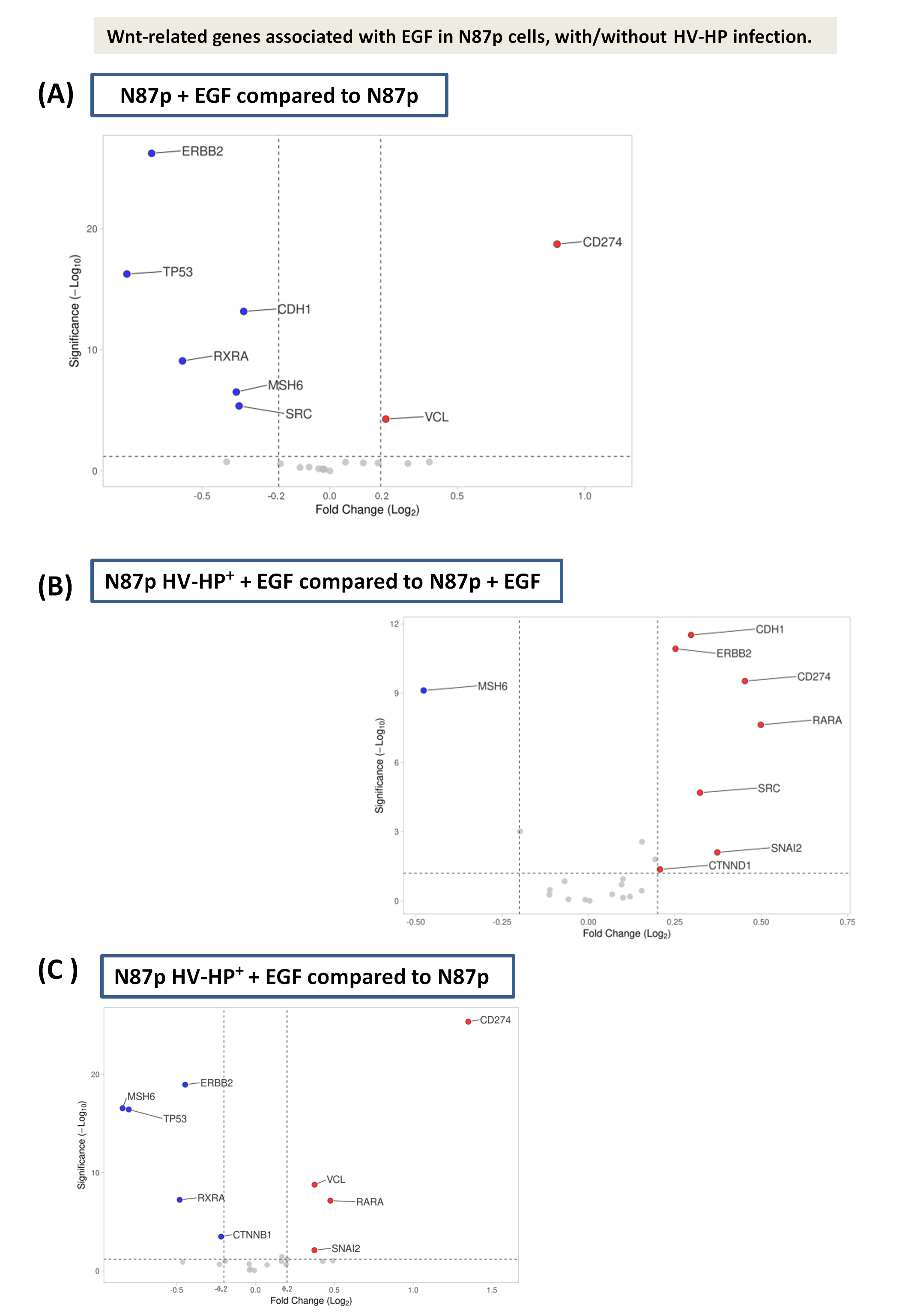

Supplement: Supplementary Figure 5 — Differential mRNA expression in EGF-treated N87p cells with or without Helicobacter pylori infection. (A) Volcano plot showing differentially expressed genes between EGF-treated and untreated N87p cells. (B) Volcano plot comparing EGF-treated N87p cells with versus without infection by a highly virulent H. pylori strain (HV-HP). (C) Volcano plot comparing EGF-treated, HV-HP-infected N87p cells to untreated and uninfected controls. Red and blue dots represent significantly upregulated and downregulated genes, respectively (p ≤ 0.05 and |log2FC| ≥ 0.2). [file Image10.tif]

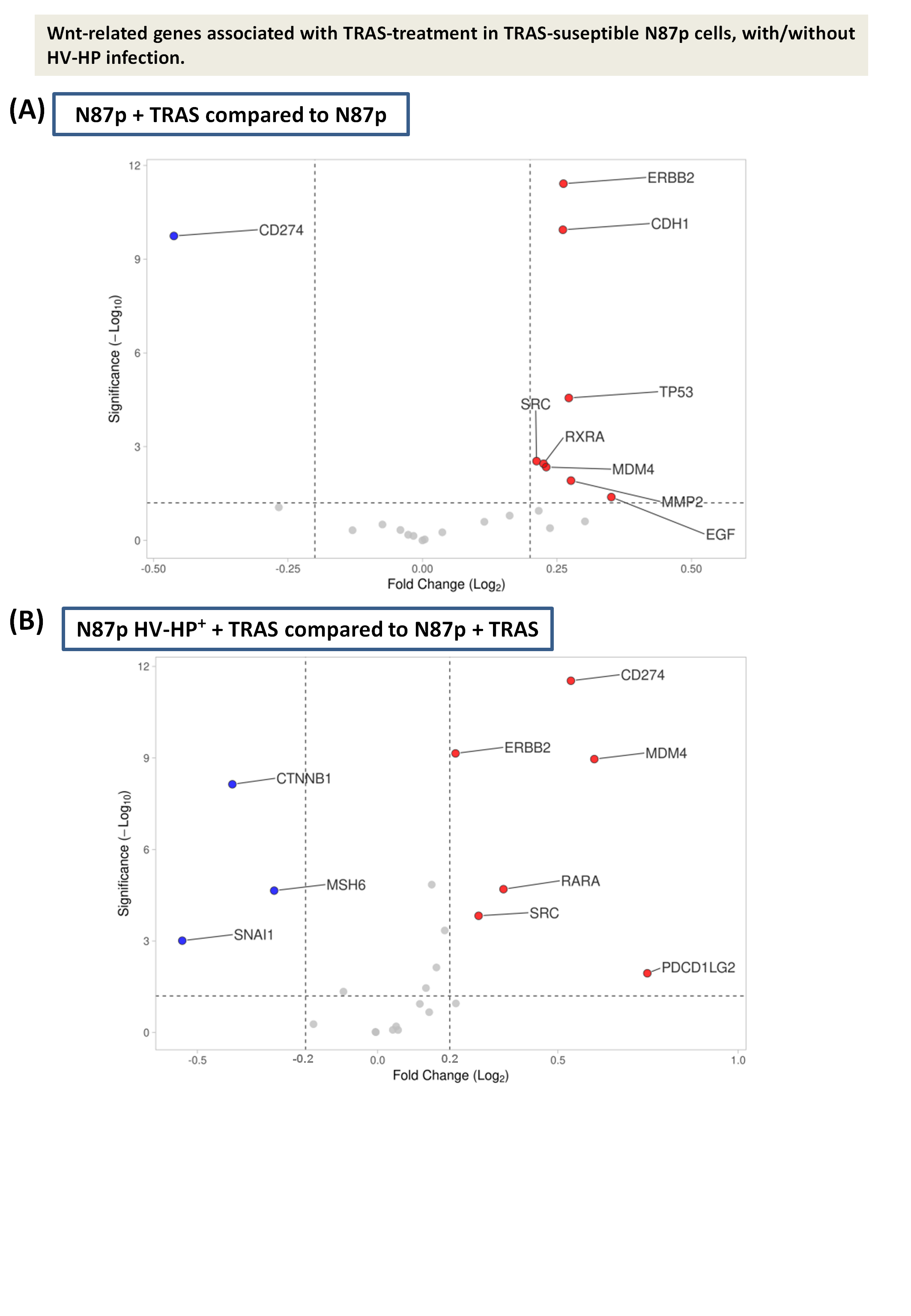

Supplement: Supplementary Figure 6 — TRAS treatment and HV-HP infection of TRAS-sensitive N87p cells. (A) Volcano plot of differentially expressed genes in TRAS-treated versus untreated N87p cells. (B) Volcano plot of HV-HP infected, TRAS-treated N87p compared to their uninfected counterpart. Red and blue dots indicate significantly upregulated and downregulated genes, respectively (p-value ≤ 0.05 and |log2FC| ≥ 0.2). [file Image11.tif]

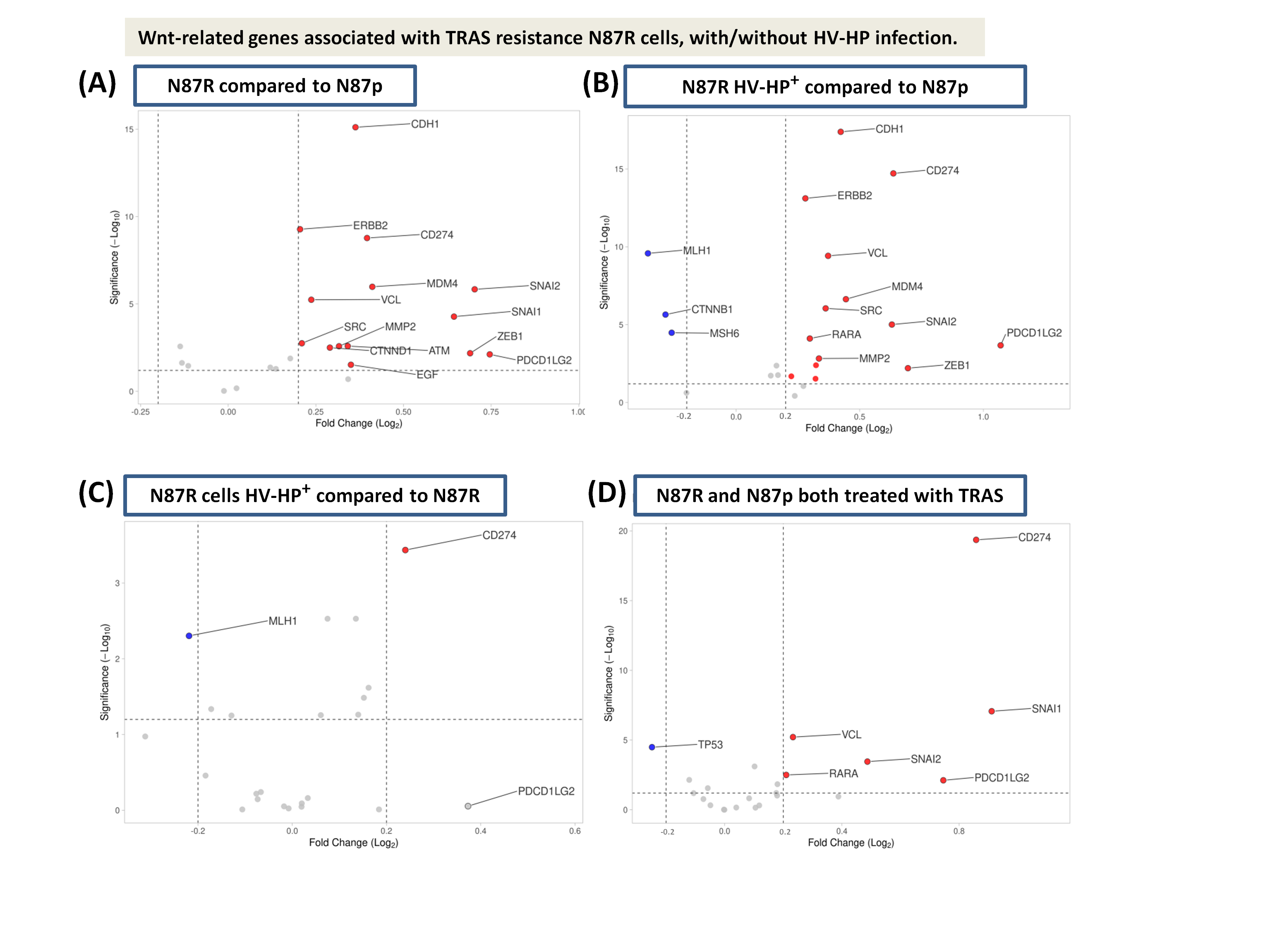

Supplement: Supplementary Figure 7 — Wnt-related genes associated with TRAS-resistance N87R cells, with or without HV-HP infection. (A) Volcano plot analysis of differentially expressed Wntβ-catenin-related genes in TRAS-resistant N87R versus TRAS-sensitive N87p cells. (B) Volcano plots highlighting differentially expressed genes in HV-HP infected N87R cells compared to uninfected N87p cells. (C) Volcano plots of differentially expressed genes in TRAS-resistant N87R with versus without HV-HP infection. (D) Volcano plots of differentially expressed genes in N87R and N87p cells, both treated with TRAS.Volcano plots show genes with an absolute log2 fold change (|log2FC|) ≥ 0.2 and p-value ≤ 0.05. The names of significantly modulated genes are labeled. HV-HP = highly virulent H. pylori strain. [file Image12.tif]

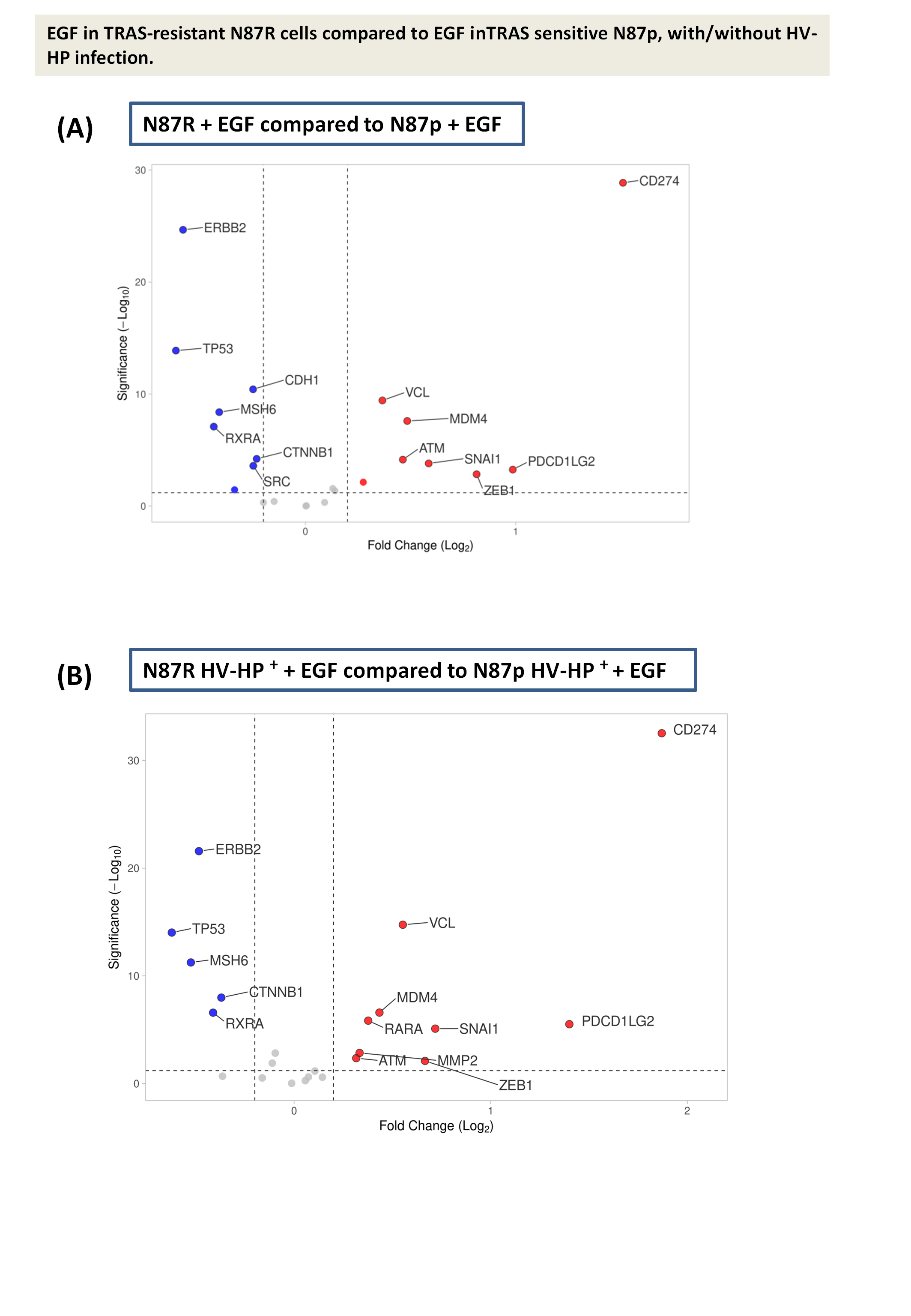

Supplement: Supplementary Figure 8 — Effects of EGF administration in TRAS-resistant N87R cells compared to TRAS-sensitive N87p cells. (A) Volcano plot comparing EGF-treated N87R cells to untreated, TRAS-sensitive N87p cells (without HV-HP infection). (B) Volcano plot comparing EGF-treated N87R cells to untreated N87p cells in the presence of HV-HP infection. Red and blue dots indicate significantly upregulated and downregulated genes, respectively (p-value ≤ 0.05 and |log2FC| ≥ |≥ 0.2). [file Image13.tif]
