# Supplementary material for: The influence of H. pylori infection in HER2-positive gastric cancer cell lines: insights from Wnt/β-catenin pathway
Source: Front Immunol. 2025 Jun 26;16:1550651. doi: 10.3389/fimmu.2025.1550651 (PMC12240786; doi:10.3389/fimmu.2025.1550651)
Supplement: Supplementary file 9 [file Table1.docx]

**Supplementary Table 1**

| Probe name | Analyte | Accession number | Annotations |
| --- | --- | --- | --- |
| ATM | mRNA | NM_000051.3 | Endogenous |
| CD274 | mRNA | NM_014143.3 | Endogenous |
| CDH1 | mRNA | NM_004360.3 | Endogenous |
| CTNNB1 | mRNA | NM_001904.3 | Endogenous |
| CTNND1 | mRNA | NM_001331.2 | Endogenous |
| EGF | mRNA | NM_001963.4 | Endogenous |
| ERBB2 | mRNA | NM_004448.2 | Endogenous |
| KLRG1 | mRNA | NM_001329099.1 | Endogenous |
| MDM4 | mRNA | NM_001204172.1 | Endogenous |
| MLH1 | mRNA | NM_000249.2 | Endogenous |
| MMP2 | mRNA | NM_001127891.1 | Endogenous |
| MSH6 | mRNA | NM_000179.2 | Endogenous |
| PDCD1LG2 | mRNA | NM_025239.3 | Endogenous |
| RARA | mRNA | NM_001033603.1 | Endogenous |
| RPL19 | mRNA | NM_000981.3 | Endogenous |
| RXRA | mRNA | NM_002957.4 | Endogenous |
| SNAI1 | mRNA | NM_005985.2 | Endogenous |
| SNAI2 | mRNA | NM_003068.4 | Endogenous |
| SRC | mRNA | NM_005417.3 | Endogenous |
| TP53 | mRNA | NM_001126118.1 | Endogenous |
| VCL | mRNA | NM_014000.2 | Endogenous |
| WNT1 | mRNA | NM_005430.3 | Endogenous |
| WNT7A | mRNA | NM_004625.3 | Endogenous |
| ZEB1 | mRNA | NM_001128128.1 | Endogenous |
| ACTB | mRNA | NM_001101.2 | Housekeeping |
| B2M | mRNA | NM_004048.2 | Housekeeping |
| GAPDH | mRNA | NM_001256799.1 | Housekeeping |
| HPRT1 | mRNA | ENST00000298556.8 | Housekeeping |
| RPL27 | mRNA | NM_000988.4 | Housekeeping |
| TBP | mRNA | NM_001172085.1 | Housekeeping |
